# A-to-I RNA editing remodels 5′-UTR initiation codons to tune translational output

**DOI:** 10.1038/s41598-026-57909-0

**Published:** 2026-06-17

**Authors:** Yuki Ogata, Asuka Ichinomiya, Momoko Tomikura, Masatora Fukuda

**Affiliations:** Department of Chemistry, Faculty of Science, Fukuoka University, Fukuoka, 814-0180 Japan

**Keywords:** RNA editing, Post-transcriptional regulation, Translational control, ADAR, Biochemistry, Molecular biology

## Abstract

A-to-I RNA editing is a prevalent post-transcriptional modification in higher eukaryotes that converts adenosine to inosine within RNA molecules. Because inosine is interpreted as guanosine during translation, editing can alter codon identity and potentially influence translation initiation signals. Here, we examined whether A-to-I editing within the 5′ untranslated region (5′-UTR) can remodel upstream initiation codons and thereby tune downstream translation. Using luciferase-based reporter systems, we show that AUA-to-AUI editing generates an initiation-competent inosine-containing codon, whereas AUG-to-IUG editing markedly attenuates initiation and can relieve uORF-mediated repression. Quantitative in vitro and cellular assays establish the initiation hierarchy AUA < AUI < AUG, with IUG exhibiting strongly reduced initiation efficiency. Importantly, AUI-mediated upstream initiation did not behave like a canonical AUG-initiated uORF in the tested contexts; its effect on downstream ORF translation was modest and context-dependent. Transcriptome-wide bioinformatic analysis identified endogenous human transcripts whose 5′-UTRs harbor editing sites compatible with initiation-codon gain or attenuation. Reporter validation using native 5′-UTR sequences supports the possibility that editing-dependent initiation-codon remodeling can tune translational output in living cells, particularly through AUG-to-IUG-mediated derepression. Together, these findings establish a reporter-based framework in which A-to-I editing can remodel 5′-UTR initiation codons, while highlighting the need for endogenous protein-level and native-locus validation to determine physiological relevance.

**Supplementary Information:**

The online version contains supplementary material available at https://doi.org/10.1038/s41598-026-57909-0.

## Introduction

A-to-I RNA editing is a post- or co-transcriptional modification in which adenosine (A) residues in RNA are enzymatically deaminated to inosine (I) by adenosine deaminases acting on RNA (ADARs)^1,2^. Because inosine is often interpreted as guanosine (G) during translation, A-to-I editing can exert functional effects equivalent to A-to-G point mutations. A classic example is the editing of the GluR2 pre-mRNA in the mammalian brain, where a single amino acid substitution induced by A-to-I editing alters the calcium permeability of AMPA receptors^3^. Beyond protein recoding, A-to-I editing plays multifaceted regulatory roles in diverse biological processes, including alternative splicing, microRNA biogenesis and targeting, and modulation of innate immune responses^4–7^.

This mechanism is evolutionarily conserved among higher eukaryotes, and more than three million A-to-I editing sites have been catalogued in the human transcriptome^8,9^. Recent computational analyses suggest that the number of potential editing sites may exceed 100 million^10^.

In recent years, A-to-I RNA editing has also emerged as a promising approach for transient and reversible modulation of gene expression. Unlike genome editing, RNA editing introduces no permanent DNA changes, providing a potentially safer strategy for therapeutic applications^11–15^. Two major strategies have been established for site-directed RNA base editing: (i) co-expression of an exogenous ADAR enzyme and a guide RNA (gRNA) to direct editing activity, and (ii) recruitment of endogenous ADARs using a gRNA alone^16–22^. Various RNA editing technologies have been developed to achieve site-directed A-to-I conversion. Among them, approaches that harness endogenous ADAR activity using only a synthetic oligonucleotide—termed RNA editing oligonucleotides (REOs)—have recently attracted considerable attention as a novel modality of nucleic acid therapeutics, and drug development efforts based on this platform are now actively underway.

Despite these advances, most applications of RNA editing technologies remain focused on correcting pathogenic G-to-A mutations associated with monogenic diseases. Elucidating novel gene regulatory mechanisms mediated by RNA editing could expand the spectrum of diseases that can be targeted by RNA editing–based therapeutics, thereby broadening its potential beyond mutation correction to dynamic control of gene expression.

The 5′ untranslated region (5′-UTR) of mRNA plays a crucial role in determining translation efficiency by influencing ribosome recruitment, scanning, and initiation. Recent findings have revealed that upstream open reading frames (uORFs) located in the 5′-UTR can function as potent cis-regulatory elements that modulate ribosomal scanning and re-initiation, thereby controlling translation efficiency of the main open reading frame (mORF)^23–25^. Translation of uORFs consumes pre-initiation complexes (PICs) and directly influences mORF expression, often in response to environmental or pathological stimuli^26,27^. Notably, insulin biosynthesis itself provides a well‑established example of how uORFs embedded in the 5′-UTR can exert strong translational control. Embryonic proinsulin mRNA contains two upstream AUG codons that initiate uORF translation and markedly attenuate translation of the downstream insulin coding sequence. Mutation of these upstream AUGs restores efficient translation, demonstrating that uORF‑mediated consumption of scanning ribosomes can serve as a potent regulatory mechanism. These findings highlight how sensitive physiological processes, such as insulin production, rely on uORF‑dependent modulation of ribosome initiation efficiency^28^.

We hypothesized that A-to-I RNA editing could extend beyond protein recoding to include dynamic modulation of translation initiation by altering start-codon recognition within 5′-UTRs. Specifically, editing of an isoleucine codon (AUA) to inosine-containing AUI could generate an initiation-competent noncanonical codon with the potential to create a weak uORF, whereas editing of an existing start codon (AUG) to IUG could attenuate translation initiation and suppress uORF formation. Because initiation from noncanonical codons is expected to be weaker and more context-dependent than initiation from AUG, we evaluated codon initiation activity and downstream mORF regulation as separable outcomes. We employed site-directed RNA editing technologies^18,29^ and reporter assays to test how uORF start-codon gain (AUA-to-AUI) or attenuation (AUG-to-IUG) affects translation of the downstream mORF. Furthermore, by integrating public RNA editing databases with genomic information, we identified candidate endogenous 5′-UTR architectures and tested selected native 5′-UTR sequences in reporter assays to assess whether editing-dependent start-codon remodeling can tune translational output in cellular sequence contexts.

## Results

### Functional characterization of RNA editing-generated AUI and IUG codons as translation initiation codons

**Fig. 1:**
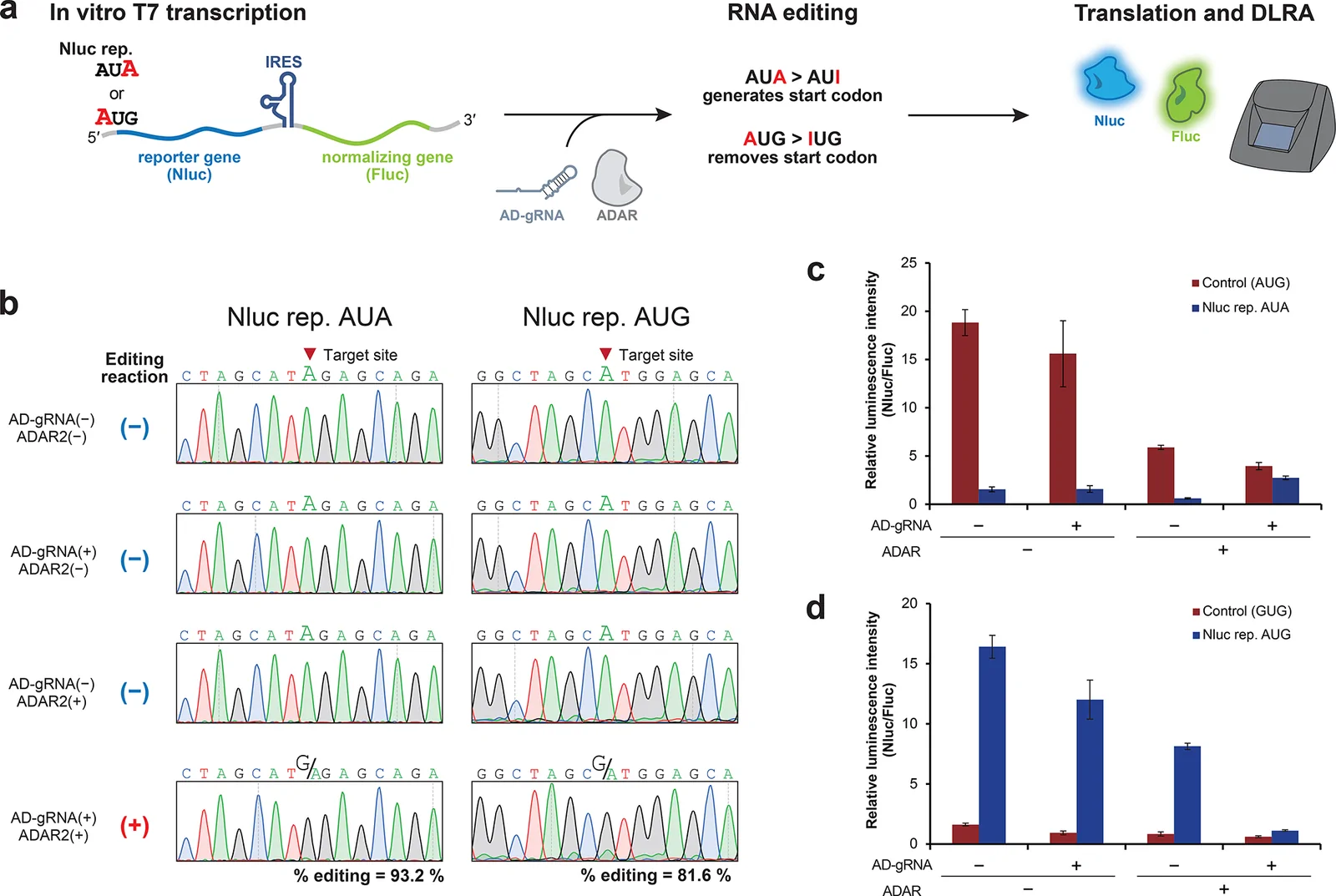
Functional characterization of RNA editing-generated AUI and IUG codons as translation initiation codons. **a** Schematic overview of the luciferase-based reporter RNA system used to quantify translation driven by A-to-I RNA editing–generated initiation codons. The Nluc reporter series consists of synthetic mRNAs encoding NanoLuc^®^ luciferase (Nluc), followed by an internal ribosome entry site (IRES) and Firefly luciferase (Fluc), which serves as an internal reporter reference for normalization within the assay. Site‑directed A‑to‑I RNA editing at the designated initiation codon was induced by recruiting ADAR to the target site via an ADAR‑guide RNA (AD‑gRNA), thereby generating reporter constructs harboring either an AUI or an IUG codon at the Nluc start site. The resulting reporter RNAs were subjected to in vitro translation reactions, and translational output was quantified using the Dual‑Luciferase Reporter Assay (DLRA). **b** Chromatograms obtained from Sanger sequencing of the regions flanking each reporter’s target site, along with the corresponding editing efficiencies calculated from peak area ratios at the edited nucleotide. The left panel illustrates whether A‑to‑I RNA editing occurred under each AD‑gRNA and ADAR combination. Red arrows indicate the target adenosine subjected to editing. **c**, **d** Relative Nluc luminescence obtained by in vitro translation followed by DLRA for each reporter RNA subjected to the editing conditions described in **b**. Values represent the mean ± standard deviation (SD) from three independent in vitro editing and translation assays.

To examine whether A-to-I RNA editing in the 5′-UTR can alter start-codon function, we first evaluated whether the edited triplets AUI and IUG can function as translation initiation codons. This experiment was designed to distinguish initiation competence of an edited codon from its subsequent ability to regulate a downstream mORF. To this end, we constructed a luciferase-based reporter RNA system to quantitatively measure the translation initiation efficiency of each codon (Fig. 1a).

The reporter mRNA (Nluc rep. series) consists of a NanoLuc^®^ luciferase (Nluc) open reading frame followed by an internal ribosome entry site (IRES) and a Firefly luciferase (Fluc) open reading frame as an internal reporter reference within the assay. In addition to the construct containing the canonical AUG start codon (Nluc rep. AUG), we prepared a variant bearing an AUA codon at the start position (Nluc rep. AUA). To generate reporter RNAs containing AUI or IUG at the initiation site, we employed a site-directed A-to-I RNA editing approach using ADAR2 and a specific ADAR guide RNA (AD-gRNA), resulting in Nluc rep. AUI and Nluc rep. IUG, respectively. The editing efficiencies for Nluc rep. AUI and Nluc rep. IUG were 93.2% and 81.6%, respectively (Fig. 1b).

Using these four reporter RNAs (AUA, AUI, AUG, and IUG), we performed in vitro translation assays and quantified Nluc luminescence as an indicator of translation initiation efficiency. AUA-to-AUI editing increased luminescence by approximately 4.5-fold, whereas AUG-to-IUG editing decreased luminescence by about 80% (Fig. 1c, d). These data show that AUI is an initiation-competent but relatively weak noncanonical start codon, whereas IUG strongly attenuates initiation relative to AUG. Thus, A-to-I editing can change start-codon identity within a reporter RNA, providing a molecular basis for testing whether such codon remodeling can influence uORF-dependent translational output.

### Asymmetric effects of uORF initiation-codon creation or attenuation on downstream translation

**Fig. 2:**
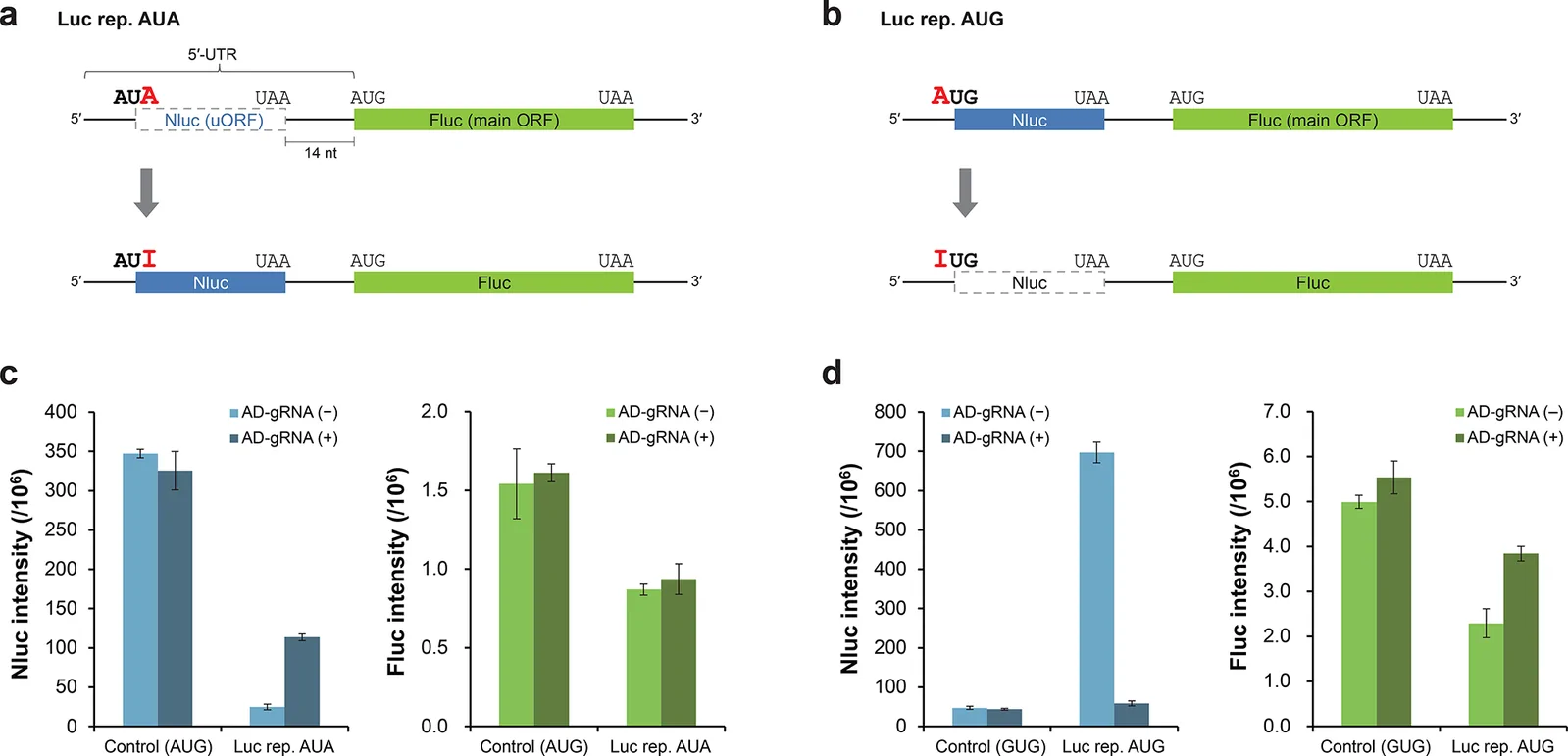
Asymmetric regulation of downstream translation by creation or attenuation of upstream initiation codons through RNA editing, **a** Schematic representation of the bicistronic luciferase reporter RNA system (“Luc rep.” series), in which the Nluc region mimics an upstream initiation/uORF proxy and the Fluc region represents the downstream mORF. In Luc rep. AUA, the Nluc start codon is replaced with AUA; subsequent A-to-I RNA editing converts AUA to AUI, thereby generating a new noncanonical upstream initiation codon. **b** Design of Luc rep. AUG, which contains a pre‑existing uORF. A‑to‑I RNA editing converts the AUG start codon to IUG, resulting in loss of uORF translation initiation. Editing efficiencies for Luc rep. AUI and Luc rep. IUG were 90.9% and 86.8%, respectively (Fig. S2). **c**, **d** Quantification of Nluc and Fluc luminescence obtained from in vitro translation-coupled dual-luciferase assays of the Luc reporter series before and after A-to-I RNA editing, generated in the presence of ADAR with or without the corresponding AD-gRNA. The left panel shows Nluc luminescence, and the right panel shows Fluc luminescence. Values represent the mean ± SD from three independent in vitro editing and translation assays.

Building on the functional characterization of AUI and IUG as translation initiation codons, we next investigated whether changes in uORF start codons within the 5′-UTR are sufficient to alter translation efficiency of the downstream main open reading frame (mORF). To test this, we designed a bicistronic luciferase reporter RNA (Luc rep. series) in which the Nluc region serves as an upstream initiation/uORF proxy and the Fluc region reports downstream ORF output (Fig. 2a). In Luc rep. AUA, the Nluc start codon was replaced with AUA; subsequent A-to-I editing converts AUA to AUI, generating a new upstream initiation site. Conversely, Luc rep. AUG contains a pre-existing uORF start codon, and A-to-I editing converts AUG to IUG, attenuating uORF translation initiation (Fig. 2b).

Using in vitro RNA editing reactions, we generated Luc rep. AUI and Luc rep. IUG with editing efficiencies of 90.9% and 86.8%, respectively (Fig. S2), and performed in vitro translation assays with each reporter RNA. Luminescence from both Nluc and Fluc was quantified to assess how editing-induced alterations in uORF initiation sites influence downstream mORF translation. In Luc rep. AUA, A-to-I editing led to an approximately 4.6-fold increase in Nluc luminescence, confirming that AUI supports upstream initiation (Fig. 2c). However, Fluc luminescence remained largely unchanged before and after editing. Thus, although AUI is initiation-competent, de novo AUI creation was not sufficient to impose strong canonical uORF-like repression on the downstream ORF in this reporter context. This result indicates that AUI should be interpreted as a weak and context-dependent upstream initiation codon rather than as a functional equivalent of AUG.

In contrast, Luc rep. AUG exhibited an approximately 12-fold reduction in Nluc luminescence following editing, with activity levels comparable to those of Luc rep. GUG, indicating that IUG strongly attenuates translation initiation. Fluc luminescence increased by approximately 1.7-fold after editing, suggesting that loss of uORF translation initiation partially relieves repression of mORF translation (Fig. 2d). The smaller magnitude of the Fluc response relative to the reduction in Nluc is consistent with the idea that downstream output is influenced not only by uORF initiation strength but also by reporter architecture, leaky scanning, reinitiation, RNA abundance, and other context-dependent factors.

Collectively, these findings reveal asymmetric consequences of A-to-I editing–mediated start-codon remodeling. AUA-to-AUI editing can generate upstream initiation, but its downstream repressive effect is weak in the tested reporter context. By contrast, AUG-to-IUG editing more effectively attenuates an existing uORF start codon and can derepress downstream translation. We therefore interpret AUI-mediated regulation as partial and context-dependent rather than as a canonical uORF logic equivalent to AUG-mediated repression.

### Sequence requirements for translational regulation by AUI/IUG editing in the 5′-UTR

To further elucidate the sequence determinants governing translational regulation by AUI and IUG editing, we next examined how the number, position, and frame organization of upstream AUG codons (uAUGs) within the 5′-UTR influence translation efficiency of the downstream mORF. As a model, we selected the 5′-UTR of the cAMP-dependent protein kinase inhibitor gamma (PKIG) gene, which contains five uAUGs. Frame annotation of this 5′-UTR showed that these five uAUGs are not five equivalent independent elements: uAUG1 and uAUG2 share a reading frame and terminate at a common UAG stop codon within the 5′-UTR, whereas uAUG3, uAUG4, and uAUG5 are in another frame; uAUG3 and uAUG4 terminate at a common UGA stop codon, while uAUG5 lies near the mORF initiation region and forms a short overlapping upstream ORF. This architecture provided a useful model to assess how specific upstream initiation codons, rather than only the total number of uAUGs, contribute to downstream translational repression.

**Fig. 3:**
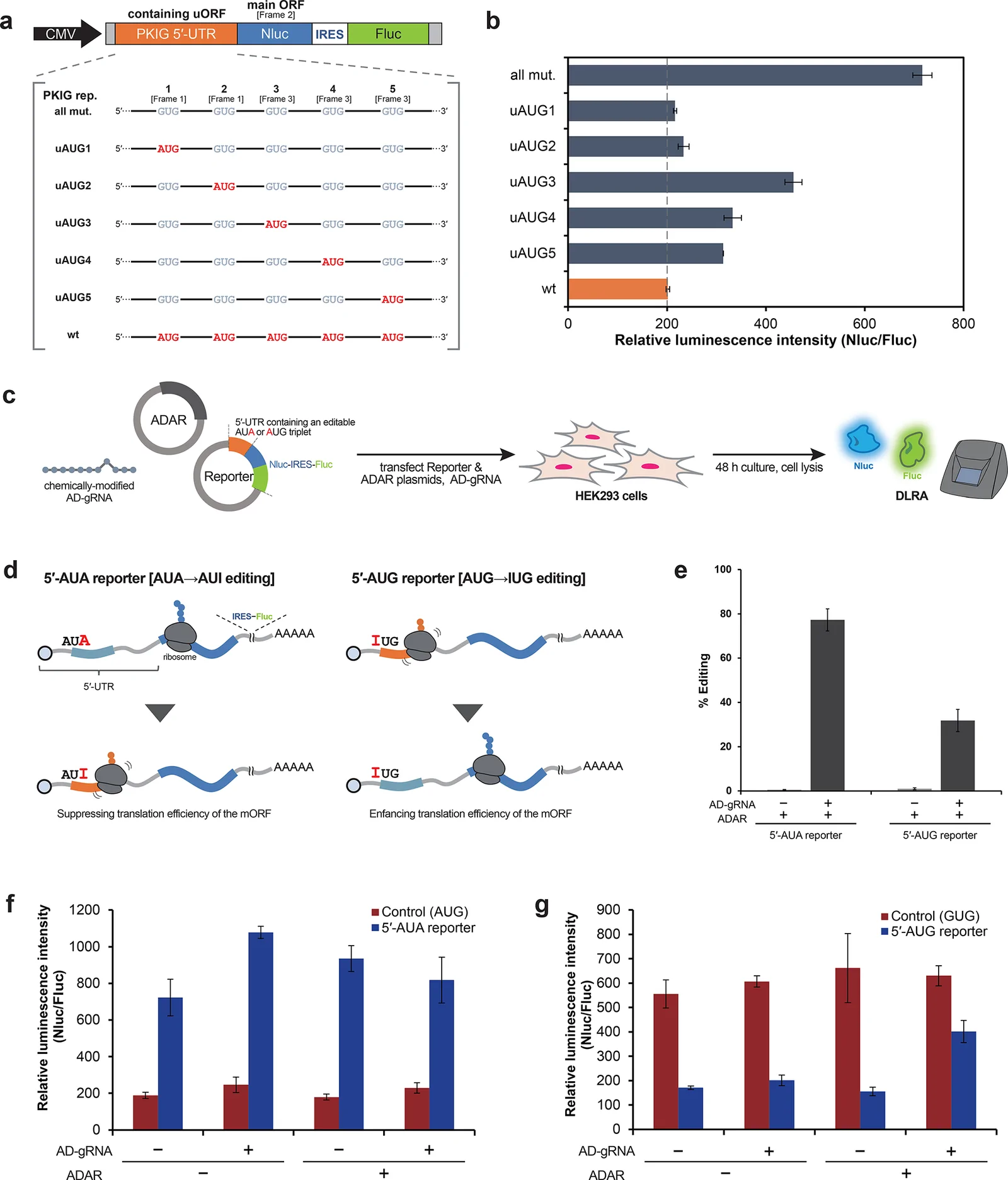
Sequence requirements for translational regulation by AUI/IUG editing in the 5′-UTR. **a** Schematic representation of the PKIG 5′-UTR reporter constructs used to evaluate how the number, position, and frame organization of upstream AUG codons (uAUGs) regulate translation of the downstream mORF. The five uAUGs are organized into two main frame groups: uAUG1 and uAUG2 share a reading frame and terminate at a common UAG stop codon, whereas uAUG3, uAUG4, and uAUG5 are in another frame; uAUG3 and uAUG4 terminate at a common UGA stop codon, and uAUG5 forms a short upstream ORF overlapping the mORF initiation region. All uAUGs within the PKIG 5′-UTR were initially substituted with GUG to generate the “PKIG rep. all mut.” construct, after which uAUGs were sequentially restored from the 5′ end to create the PKIG nAUG series (*n* = 1–5). Each PKIG-derived 5′-UTR was placed upstream of the Nluc-IRES-Fluc cassette and cloned downstream of a CMV promoter in plasmids used for expression in cultured cells. **b** Reporter expression in cultured cells transfected with plasmids encoding the indicated PKIG 5′-UTR variants. Translation efficiency was assessed by measuring luciferase activity in cell lysates. **c** Schematic of the cellular RNA editing assays using model 5′-UTR reporters designed to recapitulate weak uORF creation or uORF attenuation by A-to-I RNA editing. The “5′-AUA reporter” contains no uAUGs but includes an editable AUA triplet that can be converted to AUI, thereby generating a de novo noncanonical upstream initiation site. The “5′-AUG reporter” contains a single uAUG, which is converted to IUG upon A-to-I RNA editing, thereby attenuating uORF initiation. Site-directed editing was induced by co-transfection of reporter plasmids with hyperactive ADAR2 (ADAR2_E488Q) and chemically modified guide RNAs that recruit ADAR2 to the target site. **d** Schematic representation of the predicted translational regulatory mechanisms mediated by AUA‑to‑AUI editing in the 5′‑AUA reporter (left) and by AUG‑to‑IUG editing in the 5′‑AUG reporter (right). **e** Editing efficiencies at the target sites of the 5′‑AUA and 5′‑AUG reporters extracted from HEK293 cells following transfection. Editing analysis was performed on sample sets characterized by AD‑gRNA (–/+) under conditions of ADAR overexpression. **f**, **g** Relative Nluc luminescence of the 5′-AUA and 5′-AUG reporters, showing the translational regulation resulting from RNA editing within the 5′-UTR. Luminescence was measured in cell lysates using the DLRA under the editing conditions shown in Fig. 3e. Data are shown as mean ± SD (*n* = 3 independent biological experiments; independent transfections processed separately).

We systematically replaced all uAUGs within the PKIG 5′-UTR with GUG to generate a construct termed “PKIG rep. all mut.,” and subsequently restored uAUGs sequentially from the 5′ end to produce the PKIG nAUG series (*n* = 1–5) (Fig. 3a). Expression plasmids encoding each reporter RNA were transfected into cultured cells, and translation efficiency was assessed by measuring luminescence from cell lysates. Complete substitution of all uAUGs with GUG led to an approximately 3.5-fold increase in mORF translation compared with the wild-type (wt) construct, confirming translational repression by the PKIG uAUG architecture. Comparative analysis among the wt and sequentially restored constructs revealed that the wt, uAUG1, and uAUG2 constructs showed similarly low luminescence (Fig. 3b). Because uAUG1 and uAUG2 share the same reading frame and stop codon, similar behavior of these constructs is expected and indicates that the contribution of individual uAUGs cannot be interpreted independently of their frame organization. These results suggest that translational repression is driven by specific uAUG contexts and overlapping uORF architecture, rather than simply by the cumulative number of upstream initiation codons. Therefore, AUA-to-AUI editing in 5′-UTRs lacking strong pre-existing uAUGs, or AUG-to-IUG editing in 5′-UTRs containing a dominant uAUG, is expected to exert the clearest regulatory effect on mORF translation.

Building upon these observations, we next investigated whether such RNA editing–mediated regulation of translation operates in living cells. To this end, we designed two model reporter constructs, the “5′-AUA” and “5′-AUG” reporters, to recapitulate uORF formation and elimination via A-to-I editing (Fig. 3c). The 5′-AUA reporter lacks uAUGs but contains an AUA triplet that can be edited to AUI, thereby generating a de novo uORF. In contrast, the 5′-AUG reporter contains a single uAUG, which upon editing to IUG is predicted to abolish uORF translation and enhance mORF expression (Fig. 3d).

To induce RNA editing in cultured cells, expression plasmids encoding each reporter were co-transfected with a hyperactive ADAR2 mutant (ADAR2_E488Q)^30^ and chemically modified guide RNAs designed to direct site-directed editing (Fig. 3c). Total RNA was extracted from HEK293 cells after transfection, and the editing efficiencies at the target sites were determined to be 77.3% for the 5′-AUA reporter and 31.8% for the 5′-AUG reporter (Fig. 3e).

Under these conditions, luminescence measurements revealed that AUA-to-AUI editing in the 5′-AUA reporter led to only a slight decrease in relative luminescence (Fig. 3f). This result indicates that, although AUI can support upstream initiation, AUI creation alone is not sufficient to produce robust downstream repression in this cellular reporter. We initially considered whether the weak AUI-mediated repression in the Nluc-based assay reflected the presence of additional AUG codons within the Nluc sequence. However, even in the 5′-AUA reporter, which lacks extra AUG codons within the 5′-UTR, repression remained modest. These findings suggest that stronger AUI-mediated repression likely requires auxiliary features such as favorable position within the 5′-UTR, Kozak context, local RNA structure, or cellular conditions that alter start-codon selection. Conversely, AUG-to-IUG editing in the 5′-AUG reporter resulted in a marked increase in luminescence, consistent with attenuation of uORF-mediated translational repression (Fig. 3g). These findings are consistent with in vitro reporter assays using the Luc rep. AUG construct (Fig. 2d) and support the conclusion that AUG-to-IUG editing has a stronger derepressive effect than AUA-to-AUI editing has a repressive effect in the tested cellular context.

Collectively, these results support a sequence- and context-dependent framework in which A-to-I RNA editing in the 5′-UTR can tune protein translation by altering upstream initiation codons. Importantly, the two directions of editing are not functionally symmetric: AUA-to-AUI editing creates a weak noncanonical upstream initiation site with limited downstream repression, whereas AUG-to-IUG editing attenuates an existing uORF start codon and more robustly increases mORF output.

### Identification of endogenous transcript architectures compatible with AUI/IUG editing-dependent translational tuning

Following the reporter-based validation of editing-dependent start-codon remodeling, we next examined whether similar 5′-UTR architectures exist in endogenous human transcripts and whether such regulation could, in principle, occur naturally or be induced programmably.

To this end, we conducted bioinformatic analyses to identify transcripts that potentially undergo two distinct types of A-to-I RNA editing events within the 5′-UTR:

(i) AUA-to-AUI editing, which may generate a novel translation initiation codon.
(ii) AUG-to-IUG editing, which may abolish an existing uORF start codon.

Human protein-coding transcript sequences were retrieved from the Ensembl database^31^, yielding a total of 91,902 transcripts for analysis. Among these, 40,683 transcripts lacked any AUG codons in the 5′-UTR, and among these, 7524 contained at least one AUA triplet, suggesting the potential formation of novel start codons through AUA-to-AUI editing. These transcripts therefore represent gene groups whose translation could, in principle, be regulated through RNA editing–mediated alteration of start codon identity. In contrast, 51,219 transcripts contained at least one AUG codon within their 5′-UTR, and 17,194 transcripts contained only a single AUG codon, representing potential candidates for uORF elimination via AUG-to-IUG editing.

**Fig. 4:**
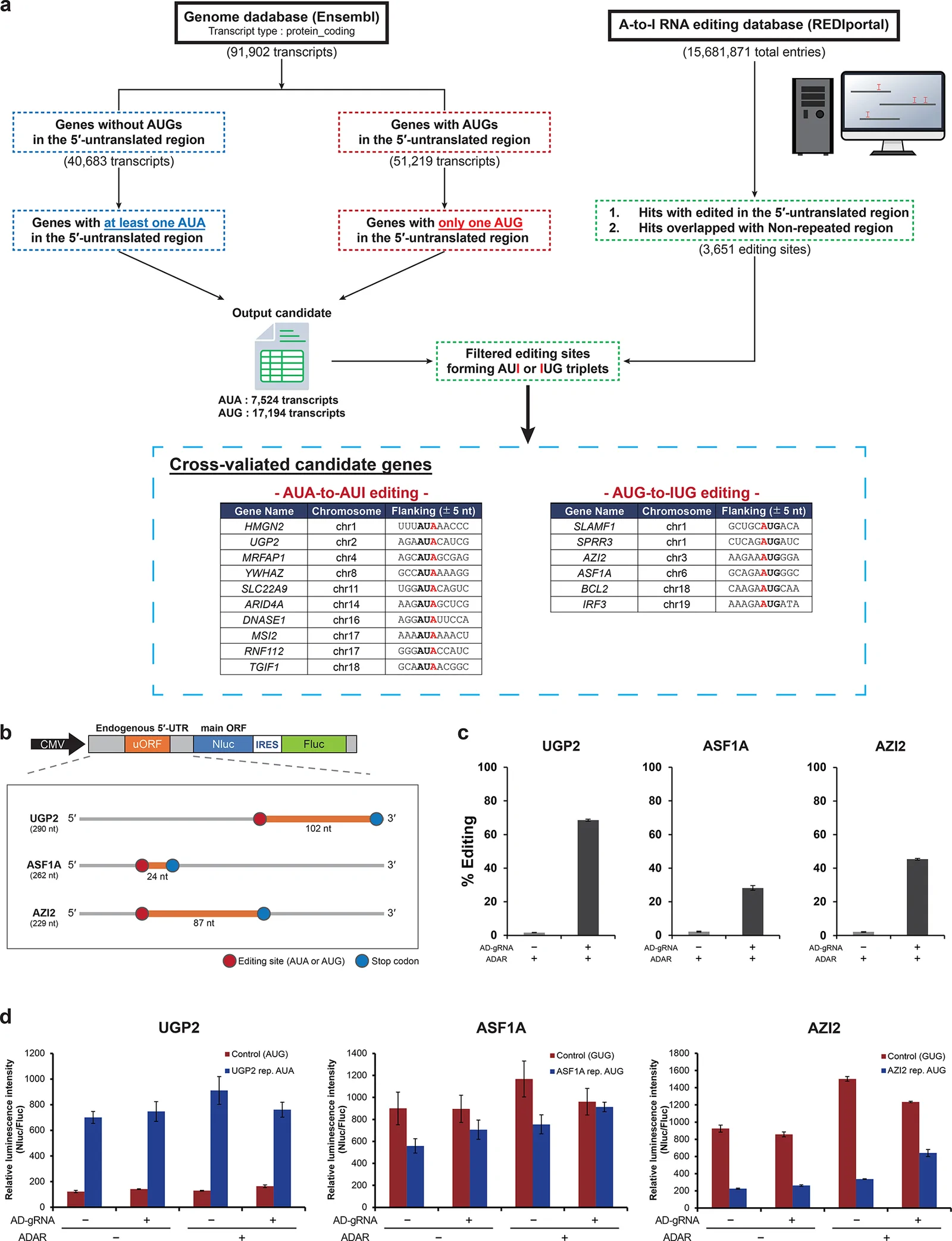
Identification and reporter validation of endogenous 5′-UTR architectures compatible with AUA-to-AUI and AUG-to-IUG editing. **a** Transcriptome-wide bioinformatic screening of human protein-coding transcripts (Ensembl; 91,902 transcripts) to identify 5′-UTR architectures potentially susceptible to A-to-I RNA editing-mediated start-codon gain or attenuation. A total of 40,683 transcripts lacked AUG codons in the 5′-UTR, among which 7,524 contained ≥ 1 AUA triplet, representing candidates for de novo noncanonical start-codon generation via AUA-to-AUI editing. In addition, 51,219 transcripts contained ≥ 1 AUG codon, including 17,194 with a single AUG, representing candidates for uORF attenuation via AUG-to-IUG editing. Cross-referencing with REDIportal identified 10 candidate genes for AUA-to-AUI-mediated repression and 6 candidate genes for AUG-to-IUG-mediated derepression. Further details of the analysis pipeline are provided in the Methods section. **b** Schematic of reporter constructs carrying native endogenous 5′-UTR sequences to test editing-dependent translational tuning in native sequence contexts. For each gene, the length of the 5′-UTR, the position of the editing site (AUA or AUG), the location of the in-frame stop codon that would be encountered if translation were initiated from the triplet containing the editing site, and the distance between these features are shown. The UGP2 5′-UTR (no AUGs, contains an AUA) models de novo weak upstream initiation by AUA-to-AUI editing, whereas the ASF1A and AZI2 5′-UTRs (each containing a single AUG) model uORF attenuation by AUG-to-IUG editing. Native 5′-UTRs were inserted upstream of the luciferase coding region in reporter RNAs/plasmids as indicated. **c** Editing efficiencies at the target adenosines in HEK293 cells upon co-transfection of an ADAR2_E488Q mutant plasmid together with target-specific AD-gRNAs that recruit the enzyme to each site. Editing levels determined by Sanger sequencing were 68.6% (UGP2), 28.2% (ASF1A), and 45.4% (AZI2). Local bystander editing analysis of nearby non-target adenosines in these target amplicons is shown in Supplementary Fig. S8. **d** Relative reporter luminescence measuring translational output under the editing conditions described in **c**. The data show the effects of site-directed editing at the designated positions within the UGP2, ASF1A, and AZI2 5′-UTRs on reporter-based translational output. Reporter activity was quantified by DLRA using cell lysates. Data are shown as mean ± SD (*n* = 3 independent biological experiments; independent transfections processed separately).

Next, to evaluate whether publicly catalogued A-to-I editing sites overlap AUA or AUG triplets within candidate 5′-UTRs, AUA- and AUG-containing transcript groups were cross-referenced with the A-to-I RNA editing database REDIportal^8,9^. This analysis identified genes in which adenosines within AUA or AUG triplets are annotated as edited or potentially editable by endogenous ADARs. These genes represent candidate architectures in which translational efficiency may be influenced by editing events occurring in the 5′-UTR. As a result, 10 genes were identified as candidates for potential translational repression via AUA-to-AUI conversion, and 6 genes as candidates for potential translational enhancement via AUG-to-IUG conversion. These results support the possibility that endogenous A-to-I editing could contribute to translational regulation by creating or attenuating translation initiation codons within 5′-UTRs (Fig. 4a), although functional validation at native loci remains necessary.

Although the number of editing sites that fulfilled all criteria was limited, our analysis indicates that human transcripts contain 5′-UTR architectures that are compatible with RNA editing-mediated start-codon remodeling. Thus, RNA editing may represent a candidate mechanism for translational tuning via attenuation of uORF initiation or creation of weak noncanonical upstream initiation sites. The physiological frequency and functional significance of such events, however, remain to be determined.

### Reporter analysis of native 5′-UTR sequences containing candidate AUI and IUG editing sites

To test whether A-to-I editing can tune translation in native 5′-UTR sequence contexts, we constructed luciferase reporter RNAs containing the native 5′-UTR sequences of UGP2, which lacks AUG codons and contains an AUA triplet, and of ASF1A and AZI2, each of which contains a single AUG codon (Fig. 4b).

These genes were selected based on their distinct 5′-UTR architectures representing both possible editing scenarios—generation of a novel noncanonical upstream initiation codon (UGP2) and attenuation of an existing uORF initiation codon (ASF1A, AZI2). This design enabled direct comparison of editing-mediated start-codon gain and loss within native 5′-UTR sequence contexts, but it was not intended to establish that these loci are naturally edited or physiologically regulated at the endogenous protein level.

Site-directed A-to-I RNA editing was induced in cultured HEK293 cells by co-transfection of an ADAR2_E488Q mutant plasmid together with target-specific AD-gRNAs designed to recruit the enzyme to each editing site. Editing efficiency at the target adenosines was quantified by Sanger sequencing and was found to be 68.6% for UGP2, 28.2% for ASF1A, and 45.4% for AZI2 (Fig. 4c).

We also inspected local bystander editing at non-target adenosines within the amplified 5′-UTR regions of UGP2, ASF1A, and AZI2 using the same Sanger chromatograms. No prominent A-to-G signals were detected at nearby non-target adenosines under the corresponding ADAR2_E488Q/AD-gRNA editing conditions (Fig. S8). Thus, within the regional coverage and sensitivity of direct Sanger analysis, the designed AD-gRNAs did not induce obvious local bystander editing in these native 5′-UTR reporter contexts.

Under these conditions, AUA-to-AUI editing in the UGP2 reporter was associated with a slight decrease in mORF expression, consistent with a weak and context-dependent repressive effect of the newly created upstream initiation site. This modest effect is consistent with the model reporter results and further indicates that AUI-mediated upstream initiation does not necessarily function as a strongly repressive canonical uORF start site.

Conversely, AUG-to-IUG editing in ASF1A and AZI2 disrupted the uORF start codon and was associated with increased mORF expression by approximately 1.2-fold and 2-fold, respectively (Fig. 4d). The magnitude of the response varied between 5′-UTR contexts despite measurable editing efficiencies, suggesting that downstream output depends on local sequence context, RNA structure, reporter abundance, and partial editing efficiency.

These reporter results support the possibility that A-to-I RNA editing within native 5′-UTR sequences can tune translation by altering upstream initiation codon identity. Nevertheless, because these assays used plasmid-based reporters and ADAR2/AD-gRNA overexpression, they do not demonstrate endogenous protein-level regulation at native loci. We therefore interpret UGP2, ASF1A, and AZI2 as candidate sequence contexts and programmable editing models rather than established examples of naturally occurring physiological regulation.

## Discussion

In this study, we used reporter-based experiments to examine whether A-to-I RNA editing can modulate protein translation by altering initiation codons within 5′-UTRs. The results support the conclusion that editing can remodel initiation-codon identity, but they also show that initiation competence and downstream regulatory strength must be distinguished. AUI can serve as an initiation-competent inosine-containing codon, whereas IUG strongly attenuates initiation from a canonical AUG position (Fig. 1c, d). However, the downstream consequences of these two editing events are asymmetric rather than equivalent.

Comparative analysis of luminescence indicated the hierarchy AUA < AUI < AUG, whereas initiation from IUG was strongly attenuated and comparable to that from GUG. Notably, AUI did not show full functional equivalence to AUG. In the Luc rep. AUA, 5′-AUA, and UGP2 reporter contexts, AUA-to-AUI editing increased or supported upstream initiation but produced only modest or negligible repression of the downstream ORF (Figs. 2c, 3f, 4d). We therefore define AUI as a weak/noncanonical initiation codon, not as a canonical uORF start codon equivalent to AUG. The biological or therapeutic relevance of AUI-mediated initiation-codon creation will likely depend on additional features such as Kozak context, position within the 5′-UTR, uORF length, termination position, RNA secondary structure, and cellular translation state.

This interpretation is consistent with structural and mechanistic considerations. Recent cryo-EM structural studies by Villamayor-Belinchón et al. (2024) revealed that accurate start-codon recognition involves hydrogen bonding between the guanine base of AUG and Gly8 of eIF1A’s N-terminal tail, stabilizing the initiator tRNA (tRNA_i_) through coordinated interactions with eIF5^32^. Because inosine lacks the C2 amino group of guanine, this hydrogen bond may be absent in AUI, potentially reducing PIC stabilization and decreasing initiation efficiency. In addition, uORF usage is frequently remodeled during cellular stress responses, including pathways involving eIF2α phosphorylation^26,27^. It is therefore possible that AUI- or IUG-mediated uORF remodeling behaves differently under integrated stress response conditions, where scanning, reinitiation, and start-codon selection are altered. This possibility was not tested in the present study and will require future analysis.

In contrast to the weak AUI effect, AUG-to-IUG editing consistently enhanced downstream translation across multiple reporter systems by attenuating pre-existing uORF initiation. Even so, the downstream increase was smaller than the reduction in upstream initiation, indicating that uORF disruption is only partially transmitted to mORF output. For partial editing, the measured reporter output reflects a mixed population of edited and unedited transcripts. The modest population-level changes observed under partial editing conditions therefore do not define a general quantitative relationship between editing percentage and translational output. They indicate that partial editing can produce detectable reporter changes in the tested contexts, but they do not establish whether lower editing efficiencies would be functionally meaningful. The magnitude of the response is likely to depend on codon identity, local 5′-UTR context, RNA abundance, protein stability, and cellular state. Defining whether a threshold exists, and determining what editing level would be physiologically or therapeutically meaningful, will require future dose-response experiments.

Transcriptome-wide screening identified endogenous mRNAs whose 5′-UTRs harbor adenosines that could form AUI or IUG triplets upon editing (Fig. 4a). Reporter validation using the 5′-UTRs of UGP2, ASF1A, and AZI2 showed that selected native 5′-UTR sequences can respond to programmable editing in cells. The editing efficiencies observed for ASF1A and AZI2 were lower than those achieved in optimized model reporter substrates, suggesting that native-like 5′-UTR contexts impose additional constraints on AD-gRNA accessibility and ADAR-mediated deamination. These lower efficiencies should be considered when interpreting the magnitude of translational changes. However, these experiments do not establish that the endogenous UGP2, ASF1A, or AZI2 transcripts are naturally edited at functionally meaningful levels, nor do they demonstrate changes in endogenous protein abundance. Thus, the candidate transcripts should be viewed as sequence contexts for testing the mechanism rather than as confirmed physiological or disease-associated targets.

Our mutational analysis of the PKIG 5′-UTR further emphasizes the importance of uORF architecture. The five uAUGs are distributed across two reading-frame groups, with uAUG1/uAUG2 and uAUG3/uAUG4 sharing downstream stop codons and uAUG5 positioned close to the mORF initiation region. This organization explains why single or sequential uAUG restoration may not produce simple additive effects and underscores the influence of positional and contextual features on ribosome scanning and initiation. Previous studies have shown that uORF initiation efficiency is shaped by cis-acting elements such as Kozak sequence context, uAUG position, and RNA secondary structure, as well as by trans-acting factors including RNA-binding proteins and microRNAs^33,34,35^. Under regulatory contexts such as stress conditions, including the integrated stress response (ISR), uORF utilization is often altered and translation can initiate from non-AUG codons^25,36,37^. Such contexts may modify the impact of AUI- or IUG-mediated initiation-codon remodeling^38,39,40,41^. Under the basal experimental conditions used here, however, AUI-mediated initiation was insufficient to impose strong translational repression, consistent with its weak and context-dependent initiation capacity.

Several limitations of the present study should be noted. First, luciferase output can reflect both translation initiation and RNA abundance. This is particularly relevant for experiments using chemically modified AD-gRNAs, because guide RNA-target RNA interactions may affect reporter RNA levels or recruit RNA-binding factors. Although intracellularly expressed AD-gRNAs reduced this concern in some experiments, RNA stability effects cannot be fully excluded (Fig. S6). Second, in reporter designs that use IRES-driven Fluc as an internal reference, IRES activity may itself be context-dependent. Thus, Fluc normalization should be interpreted as an internal reporter readout rather than as a perfect measure of mRNA abundance under all conditions.

Another limitation is that most functional experiments rely on plasmid-based or in vitro reporter systems. These systems are useful for isolating initiation-codon effects, but they do not fully reproduce endogenous transcript abundance, full-length UTR architecture, RNA localization, RNA-binding proteins, promoter context, or native chromosomal regulation. We did not perform endogenous protein measurements, genome engineering, or native-locus editing in this study. Therefore, the present work establishes a mechanistic reporter-based framework, while physiological relevance will require future validation using endogenous protein analysis and native-locus or naturally edited models.

The specificity of site-directed editing is another important consideration. In the native 5′-UTR reporter assays, we inspected non-target adenosines within the UGP2, ASF1A, and AZI2 target amplicons by direct Sanger chromatogram analysis and did not detect prominent local bystander editing (Fig. S8). However, this analysis was limited to the amplified target regions and has limited sensitivity for low-frequency editing events. Because ADAR2_E488Q was overexpressed in several cell-based assays, transcriptome-wide off-target editing cannot be excluded. Future studies using deep amplicon sequencing and RNA-seq will be necessary to define editing specificity and to determine whether non-target edits contribute to translational outcomes.

**Fig. 5:**
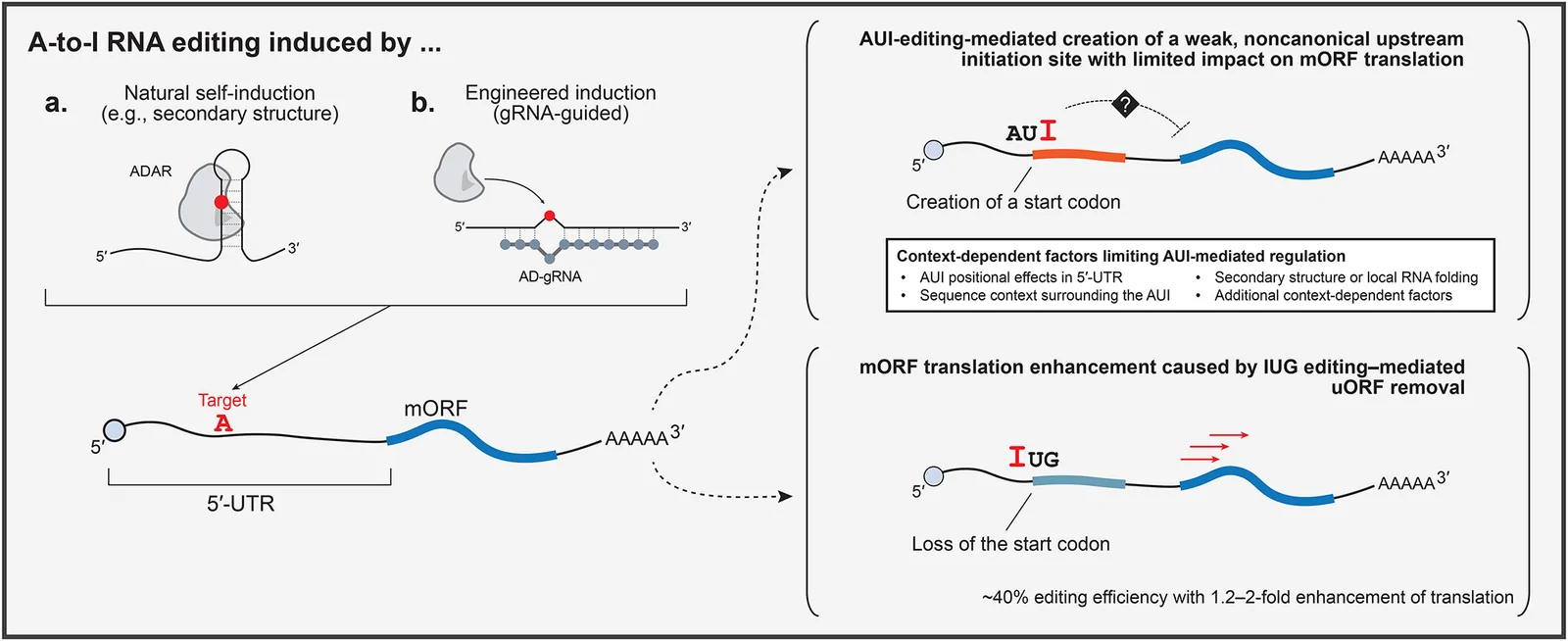
Schematic summary of context-dependent A-to-I RNA editing-mediated translational tuning proposed in this study. This figure summarizes the model proposed in this study. A-to-I RNA editing within 5′-UTRs—either naturally occurring or induced through engineered editing—can tune translation by remodeling upstream initiation codons through codon conversion (AUA-to-AUI or AUG-to-IUG). Editing-dependent acquisition of AUI generates an initiation-competent but weak noncanonical upstream initiation site; in the tested reporters, this produced only modest and context-dependent attenuation of downstream ORF translation. Conversely, editing-dependent conversion of AUG to IUG attenuates a repressive uORF start codon and enhances translation of the main ORF. Because these regulatory outcomes depend on 5′-UTR context, editing efficiency, RNA abundance, and editing specificity, endogenous protein-level validation will be required to establish physiological or therapeutic relevance.

Collectively, our findings support a model in which A-to-I RNA editing can tune translational output through 5′-UTR initiation-codon remodeling, with stronger evidence for derepression by AUG-to-IUG editing than for repression by AUA-to-AUI editing (Fig. 5). This concept is complementary to recent work such as DECOR, a TadA8e-based RNA editing platform that restored IRF6 expression by removing a pathogenic uORF in its 5′-UTR^42^. Our results suggest that the intrinsic logic of 5′-UTR initiation-codon rewiring can be exploited by programmable RNA editing (Fig. S7), but therapeutic application will require efficient and specific editing, favorable 5′-UTR context, and demonstration of endogenous protein-level benefit. Thus, editing-mediated translational tuning represents a promising but context-dependent strategy for post-transcriptional regulation rather than a universally strong bidirectional switch.

## Materials and methods

### Cell culture and transfection

HEK293 cells (ATCC) were cultured in Dulbecco’s modified Eagle’s medium (DMEM; Sigma) supplemented with 10% (v/v) fetal bovine serum (Biosera) and incubated at 37 °C with 5% (v/v) CO2. Cells were seeded in 24-well plates at a density of 1 × 10^5^ cells per well and allowed to adhere overnight. Cells were then co-transfected with a reporter plasmid (10 ng), an ADAR2_E488Q mutant expression plasmid (300 ng), and a chemically modified guide RNA (final concentration, 50 nM; modification pattern not disclosed due to intellectual property considerations) using Lipofectamine 3000 according to the manufacturer’s guidelines. Cells were harvested 48 h after transfection.

In the additional experiment described in Supplementary Figure S6, cells were transfected with a reporter plasmid (10 ng), an ADAR1 p110_E713Q mutant expression plasmid (250 ng), and an AD-gRNA expression plasmid (250 ng).

## Plasmid construction

All reporter RNA expression plasmids used in this study were generated by inserting the designated 5′-UTR reporter sequences (corresponding to each reporter name; see Supplementary Table) into either the pcDNA3.1/Hygro(–) backbone or a pre‑constructed pcDNA_IRES–Fluc backbone at the restriction enzyme sites listed in the table. The backbone and inserts were digested with the corresponding restriction enzymes supplied by TaKaRa, followed by standard restriction enzyme–ligation cloning using T4 DNA ligase (TaKaRa).

Endogenous 5′-UTR sequences were cloned based on reference transcripts retrieved from the NCBI database (PKIG: NM_001281444.2; UGP2: NM_006759.4; ASF1A: NM_014034.3; AZI2: NM_001134432.2; CNOT3: NM_014516.4). The corresponding 5′-UTR or full‑length transcript regions were amplified using PrimeSTAR GXL DNA Polymerase (TaKaRa), joined to the downstream sequence by PCR assembly, and subsequently inserted into the NheI–BamHI sites as described above. cDNA derived from SH‑SY5Y cells (ATCC) was used as the template for the PKIG 5′-UTR, whereas HEK293 cell cDNA served as the template for all other transcripts.

Mutant variants of the PKIG reporters, as well as the AUG‑ or GUG‑control reporters for the other constructs, were generated either by primer‑dependent PCR mutagenesis using the parental plasmids as templates—performed with PrimeSTAR GXL DNA Polymerase (TaKaRa)—or by synthesizing partial fragments from oligonucleotides with Klenow polymerase (New England BioLabs) and assembling them by PCR. All plasmids constructed in this study were verified by conventional Sanger sequencing to confirm sequence integrity. The sequencing was performed solely for plasmid construct validation and did not generate high-throughput DNA or RNA sequencing datasets.

## Preparation of RNA

To synthesize the target reporter RNAs, DNA templates for in vitro transcription were first generated by PCR amplification of the T7 promoter and the terminal region of each reporter sequence from the corresponding pcDNA3.1/Hygro(–) plasmid harboring the same reporter insert. For AD‑gRNA, transcription templates were assembled from synthetic oligonucleotides using Klenow polymerase (New England BioLabs).

PCR and Klenow‑generated products were purified by phenol–chloroform extraction followed by ethanol precipitation in the presence of sodium acetate. In vitro transcription reactions were carried out at 37 °C for 3 h using 1 µg of DNA template and the T7‑Scribe Standard RNA IVT Kit (CELLSCRIPT).

For target reporter RNAs, the transcription reactions were purified by phenol–chloroform extraction and ethanol precipitation, followed by direct purification on Micro Bio‑Spin P‑30 columns (Bio‑Rad) without polyacrylamide gel purification. For AD‑gRNA, the transcription products were purified by phenol-chloroform extraction and ethanol precipitation, and then resolved on a 12% denaturing polyacrylamide gel containing 8 M urea. RNA bands were recovered by the crush‑and‑soak method and precipitated with ethanol. The purified RNA was further processed using Ultrafree 0.22 μm centrifugal filters (Merck KGaA) and Micro Bio‑Spin P‑30 columns (Bio‑Rad).

Please refer to Supplementary Table for the DNA template sequences used for AD‑gRNA synthesis and the primer sets employed for generating the target reporter RNA templates.

### Synthesis of recombinant hADAR2

Recombinant ADAR2 proteins were expressed using a yeast (S. cerevisiae) expression system. DNA encoding wild-type ADAR2 (ADAR2-WT) was cloned into a yeast expression vector (pYES2/NT_A). The resulting plasmid was transformed into INVSc1 competent cells using the Frozen-EZ Yeast Transformation II Kit (ZYMO RESEARCH) and cultured on SD/−Ura agar medium (Clontech) at 30 °C for 2 days. Colonies were inoculated into 100 mL SD/−Ura broth (Clontech) and cultured in SD liquid medium (250 r/min, 30 °C) for 2 days, followed by a 24-h preculture (240 r/min, 30 °C). The preculture was transferred to 1 L expression medium (MINIMAL SD BASE GAL/RAF and − Ura DO Supplement (Clontech)) and incubated overnight (240 r/min, 30 °C). Cells were collected and washed with Buffer A [20 mM Tris-HCl, 5% glycerol, 0.5 mM DTT, 0.01% Triton X-100, 750 mM NaCl, 35 mM imidazole], supplemented with 100 U Protease Inhibitor Cocktail, and mixed with glass beads (1:1, v/v). Yeast cells were disrupted by vortexing in a cold room (4 °C) while chilled on ice (6 cycles of 5 min vortexing and 5 min incubation on ice). The lysate was purified by HisTrap HP affinity chromatography (Cytiva), and the collected fraction (1 mL) was concentrated using Amicon Ultra centrifugal filters (Ultracel-50 K; Merck KGaA). Preservation buffer [20 mM Tris-HCl, 20% glycerol, 0.5 mM DTT, 0.01% Triton X-100, 100 mM NaCl] was added to the final protein solution at a volume ratio of 1:1, and the mixture was stored at − 20 °C. Protein quantification was performed using the DC Protein Assay Kit (Bio-Rad Laboratories).

### In vitro RNA editing

The gRNA and target-RNA complexes were generated by annealing 900 nM AD-gRNA and 300 nM target-RNA by heating at 80 °C for 3 min, followed by slow cooling to 25 °C for 15 min in annealing buffer (10 mM Tris-HCl [pH 7.6] and 150 mM NaCl). After annealing, the RNA complex solution was diluted to prepare a 10 nM RNA complex. The 10 nM RNA complex and 12.5 nM or 50 nM hADAR2 were then mixed in 10 µL of reaction buffer (20 mM HEPES-NaOH [pH 7.5], 100 mM NaCl, 2 mM MgCl2, 0.5 mM DTT, 0.01% Triton X-100, 5% glycerol, 1 U/µL Murine RNase Inhibitor; New England BioLabs) and incubated at 37 °C for 1 h. RNA after reaction was purified by phenol/chloroform extraction and ethanol precipitation in the presence of sodium acetate. The purified RNA was used for RNA editing analysis experiments or in vitro translation reactions as described in the next section.

### In vitro translation

RNA (100 ng) obtained after the editing reaction was denatured at 65 °C for 5 min and annealed by rapid cooling on ice. For each translation reaction, 2.6 µL of the annealed RNA was mixed with 7.0 µL rabbit reticulocyte lysate (Promega), 0.1 µL amino acid mixture minus leucine, 0.1 µL amino acid mixture minus methionine, and 0.2 µL Murine RNase Inhibitor (New England BioLabs), and incubated at 30 °C for 1 h.

### RNA extraction and RNA editing analysis

Transfected cells were lysed in Sepasol RNA I Super G (Nacalai Tesque), and total RNA was purified by isopropanol precipitation according to the manufacturer’s instructions. Purified RNA samples were then treated with 10 U DNase I (Takara Bio) at 37 °C for 1 h, followed by phenol–chloroform extraction and ethanol precipitation. For cDNA synthesis, 0.5 µg of DNase‑treated RNA was reverse‑transcribed using an adapter‑linked oligo(dT)17 primer, 1 mM dNTPs, 1 U/µL Murine RNase Inhibitor (New England BioLabs), and PrimeScript II Reverse Transcriptase (TaKaRa). The resulting cDNA served as the template for a first PCR using the forward primer pcDNA_F01 and the reverse primer 3′‑adp, thereby amplifying reporter‑derived sequences. A second PCR targeting the predicted editing region was subsequently performed to further enrich the amplicons for downstream sequencing.

For Sanger sequencing, enriched PCR products were subjected to a Seq-PCR reaction using an editing-site-specific sequencing primer and SupreDye v3.1 (Edge Bio). The Seq-PCR products were purified by ethanol precipitation, resuspended in 15 µL Hi-Di formamide, and analyzed on an Applied Biosystems 3500 Genetic Analyzer to obtain chromatograms. A-to-I RNA editing efficiency at the target site was quantified by direct sequencing. Editing levels were calculated from the relative peak heights of A and G, using the equation: Editing ratio = G / (G + A). For local bystander editing analysis of the UGP2, ASF1A, and AZI2 5′-UTR reporter amplicons, non-target adenosines within the amplified regions were inspected in the same Sanger chromatograms under the corresponding ADAR2_E488Q/AD-gRNA editing conditions. A-to-G signals at non-target adenosines were evaluated from A and G peak patterns in the surrounding amplicon region. This analysis was used to assess prominent local bystander editing within the target amplicons, but it was not designed to sensitively detect low-frequency editing events or transcriptome-wide off-target editing.

For in vitro, purified RNA obtained as described in the in vitro RNA editing section was reverse‑transcribed using a reaction mixture containing 2.5 µM reverse primer, 1 mM dNTPs, 1 U/µL Murine RNase Inhibitor (New England BioLabs), and PrimeScript II Reverse Transcriptase (TaKaRa). The resulting cDNA was then diluted 10‑fold and used as the template for PCR amplification of the region surrounding the predicted editing site. PCR was performed using the appropriate primer set together with PrimeSTAR GXL DNA Polymerase (TaKaRa) to generate DNA fragments suitable for editing analysis. The enriched PCR products were subjected to Sanger sequencing as described above, and editing efficiencies were quantified based on the relative peak heights of A and G in the chromatograms.

Please refer to Supplementary Table for the primer sequences used in the reverse transcription reactions and PCR amplifications.

### Quantification of RNA Levels by qPCR

Following cDNA synthesis as described in the “RNA extraction” section, the resulting total RNA–derived cDNA was diluted 10-fold and subjected to quantitative PCR (qPCR) on a QuantStudio 5 Real-Time PCR System (Applied Biosystems) using Power SYBR^®^ Green PCR Master Mix (Applied Biosystems). Reporter gene–specific primers were used to quantify target transcript levels, and GAPDH was used as an internal control. qPCR cycling conditions were as follows: 95 °C for 600 s, followed by 45 cycles of 95 °C for 10 s and 60 °C for 30 s. Melting curve analysis was performed to confirm primer specificity (60 °C to 90 °C at a ramp rate of 0.1 °C/s). Relative RNA expression levels were calculated using the 2^−ΔΔCt method.

### Dual-luciferase reporter assay

For cultured cells, the medium was aspirated off, 100 µL of 1×Passive Lysis Buffer (Promega) was added per well, and the cells were detached by shaking for 15 min. Cells were then disrupted by pipetting and completely lysed. The collected cell extract was centrifuged at 4 °C, 15,000 rpm for 5 min, and 10 µL of the supernatant was aliquoted.

The Nano DLR™ Stop & Glo^®^ reagent was prepared by mixing Nano DLR™ Stop & Glo^®^ substrate and Nano DLR™ Stop & Glo^®^ Buffer (Promega) at a ratio of 1:100. For luciferase measurements, 40 µL ONE-Glo^®^ reagent was added to 10 µL of cell extract or in vitro translation product, mixed by pipetting, briefly centrifuged, and incubated for 1 min at room temperature. Firefly luciferase (Fluc) luminescence was measured using a GloMax 20/20 Luminometer (Promega). Subsequently, 40 µL Nano DLR™ Stop & Glo^®^ reagent was added to the same sample, mixed, briefly centrifuged, and incubated for 1 min, and NanoLuc (Nluc) luminescence was measured.

### Statistical analysis and replicate definition

Unless otherwise indicated, values are shown as mean ± standard deviation (SD) from three independent experiments. For in vitro assays, independent experiments refer to separately prepared RNA editing and translation reactions. For cell-based assays, independent experiments refer to independent transfections processed separately. Where pairwise statistical comparisons were performed between matched control and editing conditions processed in parallel, two-tailed paired Student’s t-tests were used. For comparisons among multiple reporter constructs, one-way analysis of variance followed by Tukey’s multiple-comparison test was used. Statistical details are indicated in the corresponding figure legends where applicable.

### Candidate gene identification

Candidate genes were first retrieved using the Ensembl BioMart web interface by filtering for protein‑coding transcripts. For each transcript, the corresponding 5′-UTR sequence and Ensembl transcript ID were extracted. The collected 5′-UTR sequences were then categorized according to the number of AUG codons present within the UTR. Based on this classification, transcripts were further sorted into the following two groups:

1. AUA‑containing 5′-UTRs lacking any AUG codons, representing candidates for AUA‑to‑AUI editing.
2. 5′-UTRs containing exactly one AUG codon, representing candidates for AUG‑to‑IUG editing.

To refine these candidate sets, we queried the REDIportal RNA editing database (Organism: Homo sapiens; Genome version: hg38; Location: NONREP; Genic region: 5′-UTR; AA change: Any; all other parameters kept at default settings). Genes exhibiting AUA-to-AUI or AUG-to-IUG editing events in REDIportal were cross-referenced with the Ensembl-derived candidates, and only transcripts present in both datasets were retained as final candidates. These transcripts represent candidate sequence contexts predicted to be compatible with A-to-I RNA editing-mediated modulation of translation through the mechanisms proposed in this study.

The information for the final candidate genes identified through these procedures is provided in Supplementary Table.

## Supplementary Information

Below is the link to the electronic supplementary material.

Supplementary Material 1

Supplementary Material 2

Supplementary Material 3

## Data Availability

All data generated or analyzed during this study are included in this published article, its Supplementary Information files, and the Source Data file (SR_SD_Ogata.xlsx). Raw Sanger sequencing chromatogram files are available from the corresponding author upon reasonable request.

## References

[1] Rueter, S. M. et al. Glutamate receptor RNA editing in vitro by enzymatic conversion of adenosine to inosine. *Science* **267**, 1491–1494 (1995). 7878468 10.1126/science.7878468

[2] Nishikura, K. Functions and regulation of RNA editing by ADAR deaminases. *Annu. Rev. Biochem.* **79**, 321–349 (2010). 20192758 10.1146/annurev-biochem-060208-105251 PMC2953425

[3] Vollmar, W. et al. RNA editing (R/G site) and flip–flop splicing of the AMPA receptor subunit GluR2 in nervous tissue of epilepsy patient. *Neurobiol. Dis.* **15**, 371–379 (2004). 15006707 10.1016/j.nbd.2003.11.006

[4] Liddicoat, B. J. et al. RNA editing by ADAR1 prevents MDA5 sensing of endogenous dsRNA as nonself. *Science* **349**, 1115–1120 (2015). 26275108 10.1126/science.aac7049 PMC5444807

[5] Wang, I. X. et al. ADAR regulates RNA editing, transcript stability, and gene expression. *Cell. Rep.* **5**, 849–860 (2013). 24183664 10.1016/j.celrep.2013.10.002 PMC3935819

[6] Hsiao, Y. E. et al. RNA editing in nascent RNA affects pre-mRNA splicing. *Genome Res.* **28**, 812–823 (2018). 29724793 10.1101/gr.231209.117 PMC5991522

[7] Yang, W. et al. Modulation of microRNA processing and expression through RNA editing by ADAR deaminases. *Nat. Struct. Mol. Biol.* **13**, 13–21 (2006). 16369484 10.1038/nsmb1041 PMC2950615

[8] Picardi, E. et al. REDIportal: a comprehensive database of A-to-I RNA editing events in humans. *Nucleic Acids Res.* **45**, D750–D757 (2017). 27587585 10.1093/nar/gkw767 PMC5210607

[9] Mansi, L. et al. REDIportal: millions of novel A-to-I RNA editing events from thousands of RNAseq experiments. *Nucleic Acids Res.* **49**, D1012–D1019 (2021). 33104797 10.1093/nar/gkaa916 PMC7778987

[10] Bazak, L. et al. A-to-I RNA editing occurs at over a hundred million genomic sites, located in a majority of human genes. *Genome Res.* **24**, 365–376 (2014). 24347612 10.1101/gr.164749.113 PMC3941102

[11] Gagnidze, K. et al. A new chapter in genetic medicine: RNA editing and its role in disease pathogenesis. *Trends Mol. Med.* **24**, 294–303 (2018). 29483039 10.1016/j.molmed.2018.01.002

[12] Gold, A., Levanon, E. Y. & Eisenberg, E. The new RNA-editing era-ethical considerations. *Trends Genet.* **37**, 685–687 (2021). 33975753 10.1016/j.tig.2021.04.013

[13] Diaz Quiroz, J. F., Siskel, L. D. & Rosenthal, J. J. C. Site-directed A → I RNA editing as a therapeutic tool: moving beyond genetic mutations. *RNA* **29**, 498–505 (2023). 36669890 10.1261/rna.079518.122 PMC10019371

[14] Booth, B. J. et al. RNA editing: Expanding the potential of RNA therapeutics. *Mol. Ther.* **31**, 1533–1549 (2023). 36620962 10.1016/j.ymthe.2023.01.005 PMC9824937

[15] Dadush, A. et al. DNA and RNA base editors can correct the majority of pathogenic single nucleotide variants. *NPJ Genomics Med.* **9**, 16 (2024). 10.1038/s41525-024-00397-w PMC10897195 38409211

[16] Stafforst, T. & Schneider, M. F. An RNA-deaminase conjugate selectively repairs point mutations. *Angew Chem. Int. Ed.* **51**, 11166–11169 (2012). 10.1002/anie.201206489 23038402

[17] Cox, D. B. T. et al. RNA editing with CRISPR–Cas13. *Science* **358**, 1019–1027 (2017). 29070703 10.1126/science.aaq0180 PMC5793859

[18] Fukuda, M. et al. Construction of a guide-RNA for site-directed RNA mutagenesis utilising intracellular A-to-I RNA editing. *Sci. Rep.* **7**, 41478 (2017). 28148949 10.1038/srep41478 PMC5288656

[19] Merkle, T. et al. Precise RNA editing by recruiting endogenous ADARs with antisense oligonucleotides. *Nat. Biotechnol.* **37**, 133–138 (2019). 30692694 10.1038/s41587-019-0013-6

[20] Reautschnig, P. et al. CLUSTER guide RNAs enable precise and efficient RNA editing with endogenous ADAR enzymes in vivo. *Nat. Biotechnol.* **40**, 759–768 (2022). 34980913 10.1038/s41587-021-01105-0

[21] Yi, Z. et al. Engineered circular ADAR-recruiting RNAs increase the efficiency and fidelity of RNA editing in vitro and in vivo. *Nat. Biotechnol.* **40**, 946–955 (2022). 35145313 10.1038/s41587-021-01180-3

[22] Sun, Y. et al. Improved RNA base editing with guide RNAs mimicking highly edited endogenous ADAR substrates. *Nat. Biotechnol.*. 10.1038/s41587-025-02628-6 (2025). 40181169

[23] Wang, X. & Rothnagel, J. A. 5′-Untranslated regions with multiple upstream AUG codons can support low‐level translation via leaky scanning and reinitiation. *Nucleic Acids Res.* **32**, 1382–1391 (2004). 14990743 10.1093/nar/gkh305 PMC390293

[24] Johnstone, T. G. et al. Upstream ORFs are prevalent translational repressors in vertebrates. *EMBO J.* **35**, 706–723 (2016). 26896445 10.15252/embj.201592759 PMC4818764

[25] Chen, J. et al. Pervasive functional translation of noncanonical human open reading frames. *Science* **367**, 1140–1146 (2020). 32139545 10.1126/science.aay0262 PMC7289059

[26] Lee, Y. et al. An upstream open reading frame regulates translation of GADD34 during cellular stresses that induce eIF2α phosphorylation. *J. Biol. Chem.* **284**, 6661–6673 (2009). 19131336 10.1074/jbc.M806735200 PMC2652341

[27] Tsang, M. J. & Cheeseman, I. M. Alternative CDC20 translational isoforms tune mitotic arrest duration. *Nature* **617**, 154–161 (2023). 37100900 10.1038/s41586-023-05943-7 PMC10461078

[28] Hernández-Sánchez, C. et al. Upstream AUGs in embryonic proinsulin mRNA control its low translation level. *EMBO J.* **22**, 5582–5592 (2003). 14532130 10.1093/emboj/cdg515 PMC213770

[29] Nose, K. et al. Short-chain guide RNA for site-directed A-to-I RNA editing. *Nucleic Acid Ther.* **31**, 58–67 (2021). 33170095 10.1089/nat.2020.0866

[30] Kuttan, A. & Bass, B. L. Mechanistic insights into editing-site specificity of ADARs. *PNAS* **109**, E3295–E3304 (2012). 23129636 10.1073/pnas.1212548109 PMC3511710

[31] Kinsella, R. J. et al. Ensembl BioMarts: a hub for data retrieval across taxonomic space. *Database* **2011**, bar030 (2011). 21785142 10.1093/database/bar030 PMC3170168

[32] Villamayor-Belinchón, L. et al. Structural basis of AUC codon discrimination during translation initiation in yeast. *Nucleic Acids Res.* **52**, 11317–11335 (2024). 39193907 10.1093/nar/gkae737 PMC11472065

[33] Medenbach, J. et al. Translational control via protein-regulated upstream open reading frames. *Cell* **145**, 902–913 (2011). 21663794 10.1016/j.cell.2011.05.005

[34] Somers, J. et al. A perspective on mammalian upstream open reading frame function. *Int. J. Biochem. Cell. Biol.* **45**, 1690–1700 (2013). 23624144 10.1016/j.biocel.2013.04.020 PMC7172355

[35] Tidu, A. et al. Critical cis-parameters influence STructure assisted RNA translation (START) initiation on non-AUG codons in eukaryotes. *NAR Genom Bioinform.* **6**, lqae065 (2024). 38863530 10.1093/nargab/lqae065 PMC11165317

[36] Chothani, S. P. et al. A high-resolution map of human RNA translation. *Mol. Cell.* **82**, 2885–2901 (2022). 35841888 10.1016/j.molcel.2022.06.023

[37] Lu, P. D. et al. Translation reinitiation at alternative open reading frames regulates gene expression in an integrated stress response. *J. Cell. Biol.* **167**, 27–33 (2004). 15479734 10.1083/jcb.200408003 PMC2172506

[38] George, C. X. et al. Expression of interferon-inducible RNA adenosine deaminase ADAR1 during pathogen infection and mouse embryo development involves tissue-selective promoter utilization and alternative splicing. *J. Biol. Chem.* **280**, 15020–15028 (2005). 15677478 10.1074/jbc.M500476200

[39] Uhlen, M. et al. Tissue-based map of the human proteome. *Science* **347**, 1260419 (2015). 25613900 10.1126/science.1260419

[40] Human Protein Atlas – ADAR1. Expression data for ADAR1 available at: https://www.proteinatlas.org/ENSG00000160710-ADAR

[41] Human Protein Atlas – ADAR2 (ADARB. 1). Expression data for ADAR2. https://www.proteinatlas.org/ENSG00000197381-ADARB1

[42] Yan, H. & Tang, W. Programmed RNA editing with an evolved bacterial adenosine deaminase. *Nat. Chem. Biol.* **20**, 1361–1370 (2024). 38969862 10.1038/s41589-024-01661-x PMC12090699

