# Supplementary material for: A-to-I RNA editing remodels 5′-UTR initiation codons to tune translational output
Source: Sci Rep. 2026 Jun 17;16:27754. doi: 10.1038/s41598-026-57909-0 (PMC13542072; doi:10.1038/s41598-026-57909-0)
Supplement: Supplementary file 1 [file 41598_2026_57909_MOESM3_ESM.pdf]

Supplementary Information

1. Chemically modified AD-gRNAs induce RNA destabilization leading to reduced Fluc expression, which is rescued by an intracellular AD-gRNA system

In cellular assays using multiple 5′-UTR reporter constructs, we observed that co-transfection with chemically modified AD-gRNAs resulted in reduced Nluc and Fluc luminescence across all reporters. Because Fluc serves as an internal control for RNA abundance, the decrease in Fluc activity suggested that reporter RNA levels were diminished under these conditions. To experimentally validate this possibility, we performed quantitative PCR (qPCR). Analysis of the AZI2 rep. AUG reporter revealed an AD-gRNA–dependent reduction in RNA abundance (Fig. S6A). Furthermore, a strong correlation was detected between Fluc luminescence and reporter RNA levels (Fig. S6B), confirming that the loss of luminescence reflects RNA destabilization rather than a direct effect on translation.

These findings raised the possibility that the chemical modifications introduced into the AD-gRNA may trigger destabilization of the target RNA through a mechanism intrinsic to chemically modified nucleic acids. To circumvent this issue, we adopted an alternative strategy in which AD-gRNAs are expressed intracellularly without chemical modifications. The stem-loop–type AD-gRNA used in the *in vitro* editing assays was cloned into the pSUPER. neo vector and co-transfected together with ADAR and reporter expression plasmids (Fig. S6C). Under these conditions, approximately 20% editing was achieved with ADAR p110_E713Q and AD-gRNA co-expression (Fig. S6D), and removal of the uAUG via RNA editing led to increased relative Nluc luminescence (Fig. S6F, right). Importantly, although the intracellularly expressed AD-gRNA exhibited lower editing efficiency than the chemically modified gRNA, it did not induce AD-gRNA–dependent reductions in Fluc luminescence or reporter RNA levels (Fig. S6E, S6F middle). These results suggest that the chemical modifications present in synthetic AD-gRNAs may elicit target RNA destabilization through a cellular mechanism not engaged by unmodified, intracellularly expressed gRNAs.

Collectively, these findings indicate that while chemical modifications are often employed to enhance editing efficiency, they may also inadvertently compromise target RNA stability. Thus, for future development and practical application of this RNA editing–based translational regulation strategy, it will be essential to optimize gRNA modification patterns not only for editing efficiency but also to avoid unintended reductions in target RNA abundance.

2. Demonstration of translational upregulation by targeting the 5′-UTR of CNOT3, a non-endogenous RNA editing substrate, supporting the generalizability of uORF-based translational regulation

Given the strong translational enhancement observed upon removal of uORFs within the 5′-UTR, we sought to evaluate the applicability of this mechanism as a generalizable strategy for translational control. Importantly, the mechanism identified in this study relies on the creation or elimination of uORFs through A-to-I RNA editing and therefore does not require the target gene to be an endogenous RNA-editing substrate. This suggests that the approach may be broadly applicable to a wide range of genes. As shown in Fig. 4, a survey of 91,902 transcripts in the Ensembl database identified 17,194 transcripts containing exactly one AUG codon within the 5′-UTR. These ~17k transcripts represent potential candidates in which uORF elimination via AUG-to-IUG editing could mediate translational upregulation (Fig. S7A).

As a pilot experiment to test this concept in a non-endogenous editing substrate, we selected the 5′-UTR of *CCR4-NOT transcription complex subunit 3* (CNOT3) from among these candidates and quantified editing-dependent changes in translation using reporter RNA and AD-gRNA, as performed in previous experiments (Fig. S7B). Editing at the target site was successfully induced by co-expression of ADAR2_E488Q and the AD-gRNA, achieving an editing level of 40.9% (Fig. S7C). Correspondingly, relative Nluc luminescence increased by approximately 1.6-fold (Fig. S7D). This degree of translational enhancement closely matches that observed with reporters derived from endogenous RNA-editing substrates, thereby reinforcing the generalizability of the uORF-based regulatory mechanism demonstrated in this study.

Supplementary Figure 1. Additional experimental details related to Fig. 1

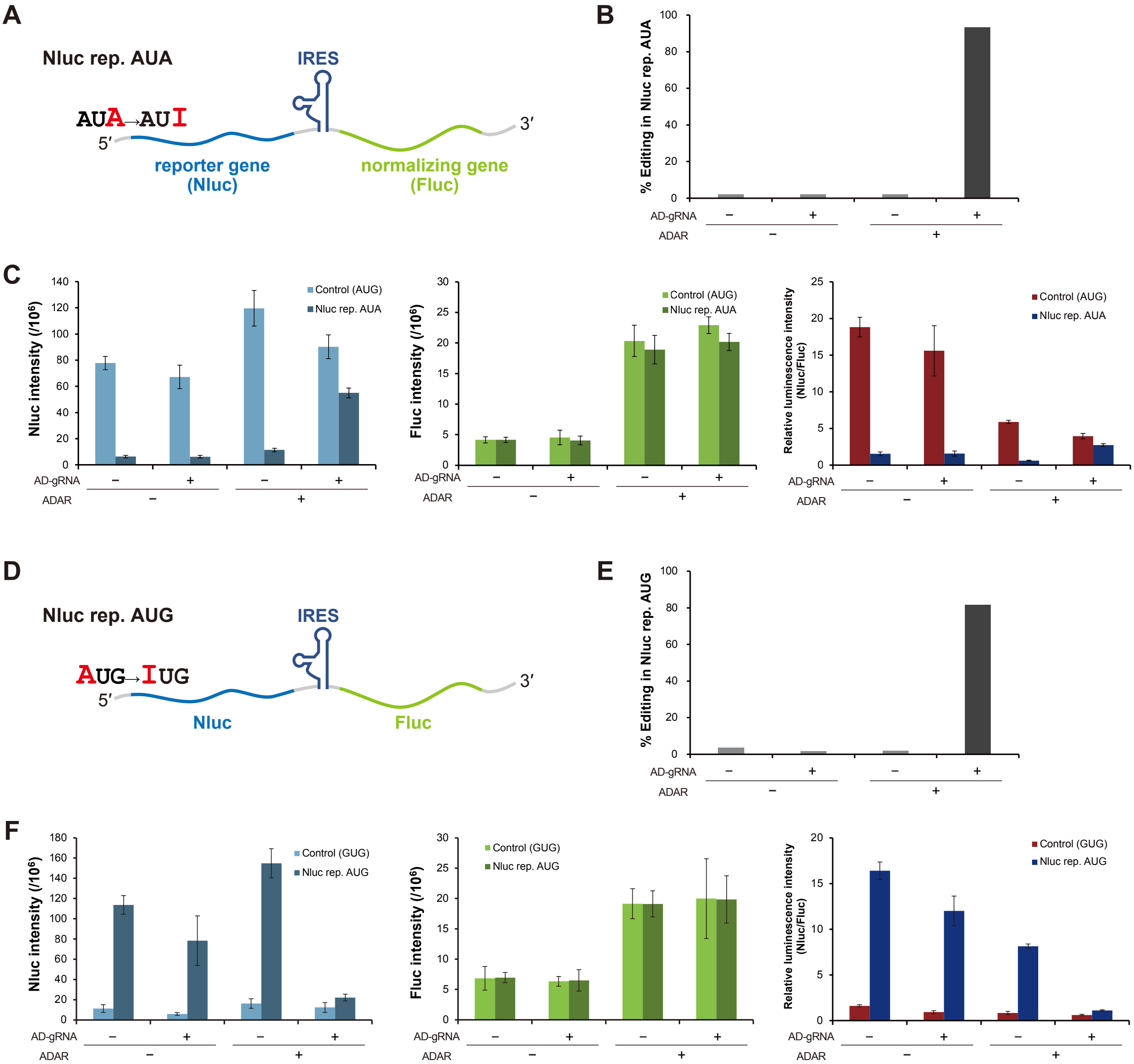

(A, D) Schematic diagrams of the reporter mRNAs used in Fig. 1. The Nluc reporter constructs consist of synthetic mRNAs encoding NanoLuc® luciferase (Nluc), followed by an internal ribosome entry site (IRES) and Firefly luciferase (Fluc), which serves as an internal control for RNA abundance. Editing of the start-codon region in Nluc generates or eliminates translation initiation sites, thereby altering Nluc translation output. These reporters were used to quantify the translational initiation potential of AUI and IUG codons based on Nluc luminescence.

(B, E) Editing efficiencies calculated from the peak area ratios of edited nucleotides in the Sanger sequencing chromatograms shown in Fig. 1b, measured for each reporter and editing condition.

(C, F) Raw luminescence values of Nluc (left) and Fluc (middle) measured under each experimental condition corresponding to Fig. 1c and 1d. These values were used to compute relative Nluc activity (right). Values represent the mean ± SD from three independent in vitro editing and translation assays. Source data are provided as a Source Data file.

Supplementary Figure 2. Additional experimental details related to Fig. 2

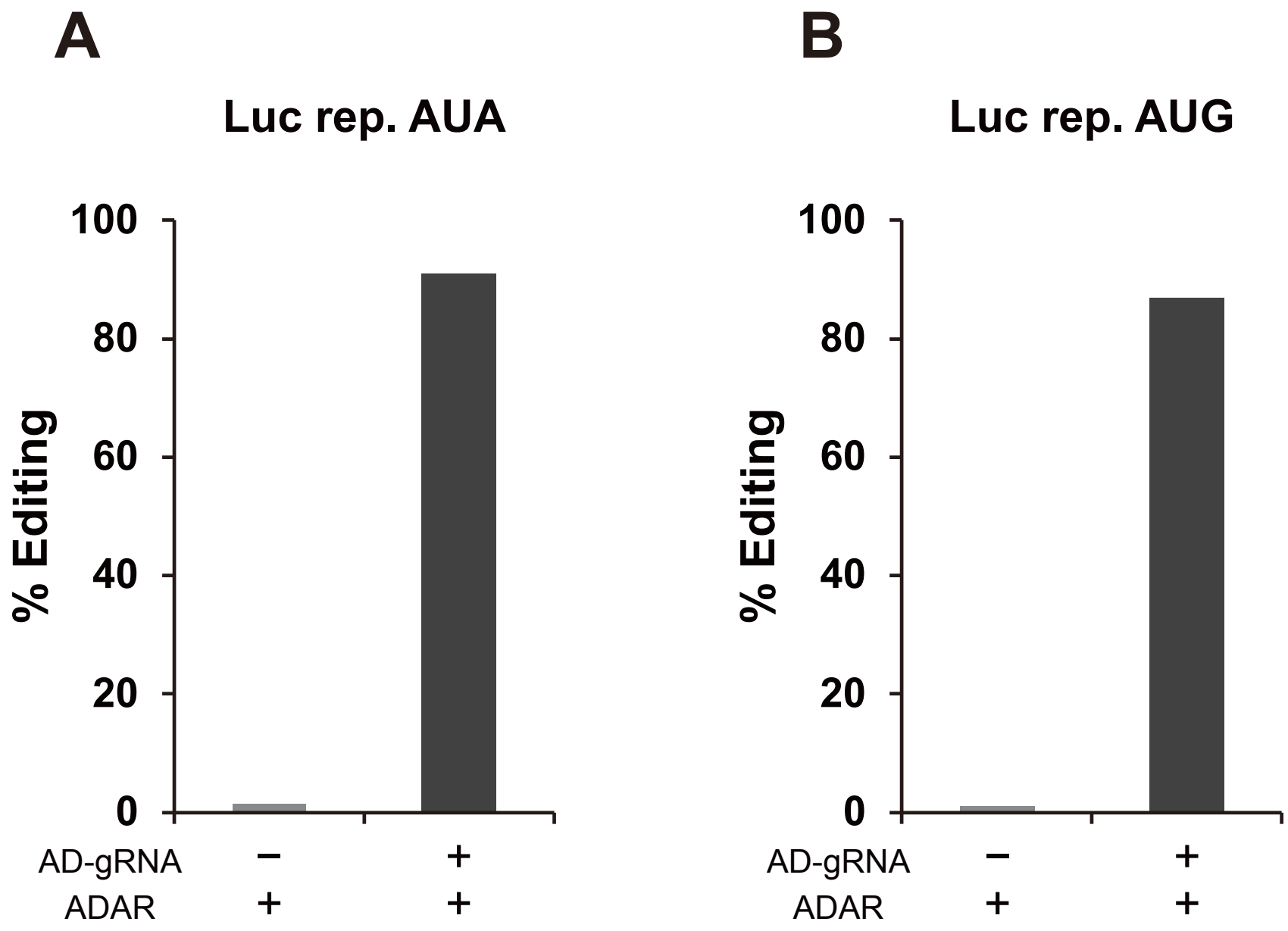

(A, B) *In vitro* RNA editing analysis of the bicistronic luciferase reporter RNAs ("Luc rep." series) used in Fig. 2, in which the Nluc start codon is engineered to mimic a uORF initiation site. Panel A shows results for the Luc rep. AUA construct, and panel B shows results for the Luc rep. AUG construct. Editing efficiencies were quantified from Sanger sequencing chromatograms by calculating the peak area ratios of the edited nucleotides under each reaction condition. Source data are provided as a Source Data file.

Supplementary Figure 3. *In vitro* evaluation of A-to-I–mediated translational regulation using 5′-AUA and 5′-AUG reporter RNAs

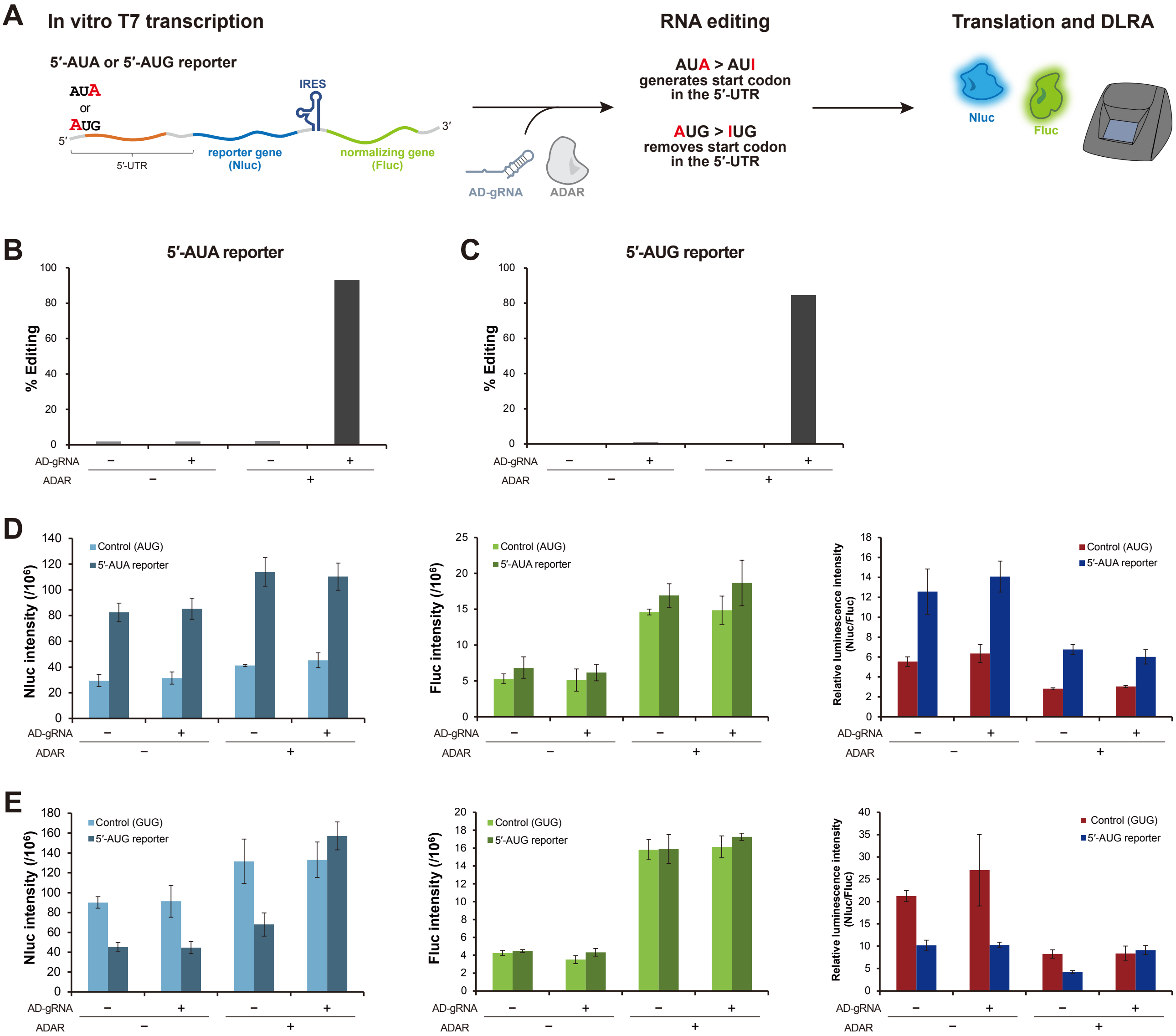

(A) Schematic overview of the luciferase-based reporter RNA system used to quantify translation regulated by A-to-I RNA editing within the 5′-UTR. The diagram illustrates the *in vitro* assay workflow for the 5′-AUA and 5′-AUG reporters analyzed in Fig. 3c–g.

(B, C) Editing efficiencies of the 5′-AUA and 5′-AUG reporters under each editing condition. Panel B shows results for the 5′-AUA reporters, and panel C shows results for the 5′-AUG reporters. Editing levels were determined from Sanger sequencing chromatograms by calculating the peak area ratios of the edited nucleotides.

(D, E) *In vitro* translation assay results corresponding to the edited RNAs generated in Fig. S3B and S3C. Panel D presents data for the 5′-AUA reporters, and panel E presents data for the 5′-AUG reporters. Shown are the raw luminescence values for Nluc and Fluc, along with the calculated relative Nluc activities. Values represent the mean ± SD from three independent in vitro editing and translation assays. Source data are provided as a Source Data file.

Supplementary Figure 4. Additional experimental details related to Fig. 3c–g

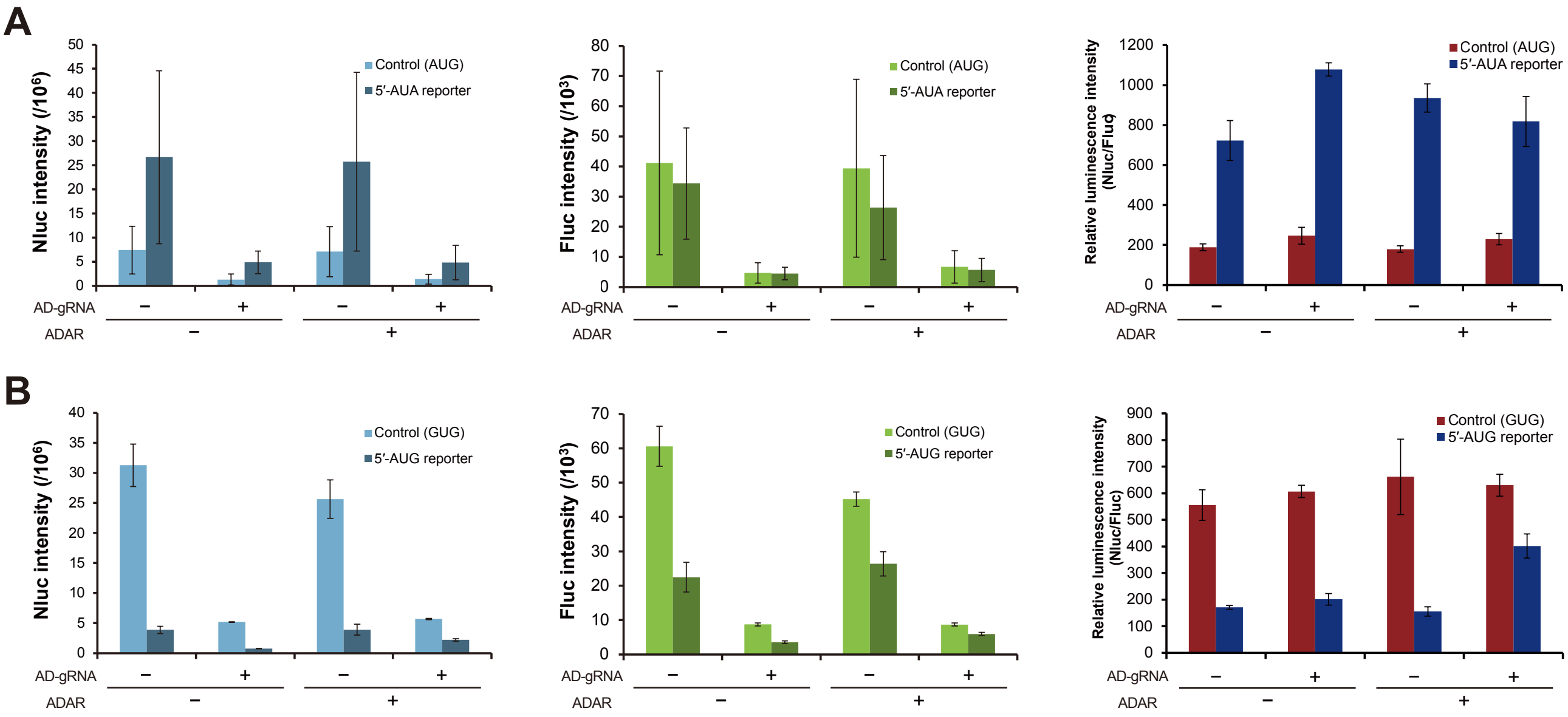

(A, B) Raw luminescence values for Nluc (left) and Fluc (middle) measured under each experimental condition corresponding to Fig. 3c–g. These values were used to compute the relative Nluc activity (right). Panel A shows data for the 5′-AUA reporters, and panel B shows data for the 5′-AUG reporters. Data are shown as mean ± SD (n = 3 independent biological experiments; independent transfections processed separately). Source data are provided as a Source Data file.

Supplementary Figure 5. Additional experimental details related to Fig. 4

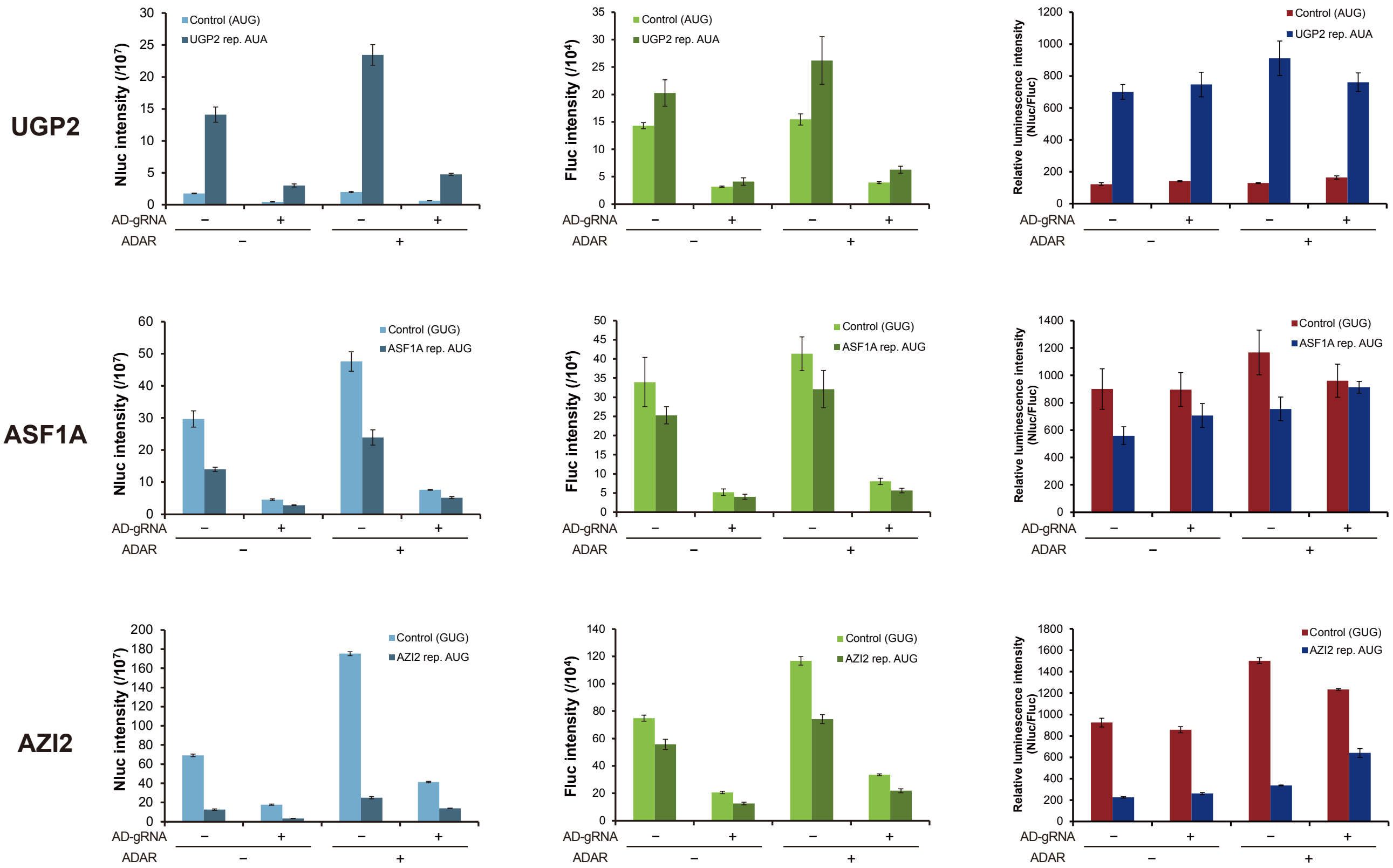

Raw luminescence values of the UGP2, ASF1A, and AZI2 reporter constructs (Nluc and Fluc) measured under each experimental condition corresponding to Fig. 4c. These raw values were used to calculate relative Nluc activity, which is shown in the right panels. Data are shown as mean ± SD (n = 3 independent biological experiments; independent transfections processed separately). Source data are provided as a Source Data file.

Supplementary Figure 6. Chemically modified AD-gRNAs induce RNA destabilization leading to reduced Fluc expression, which is rescued by an intracellular AD-gRNA system

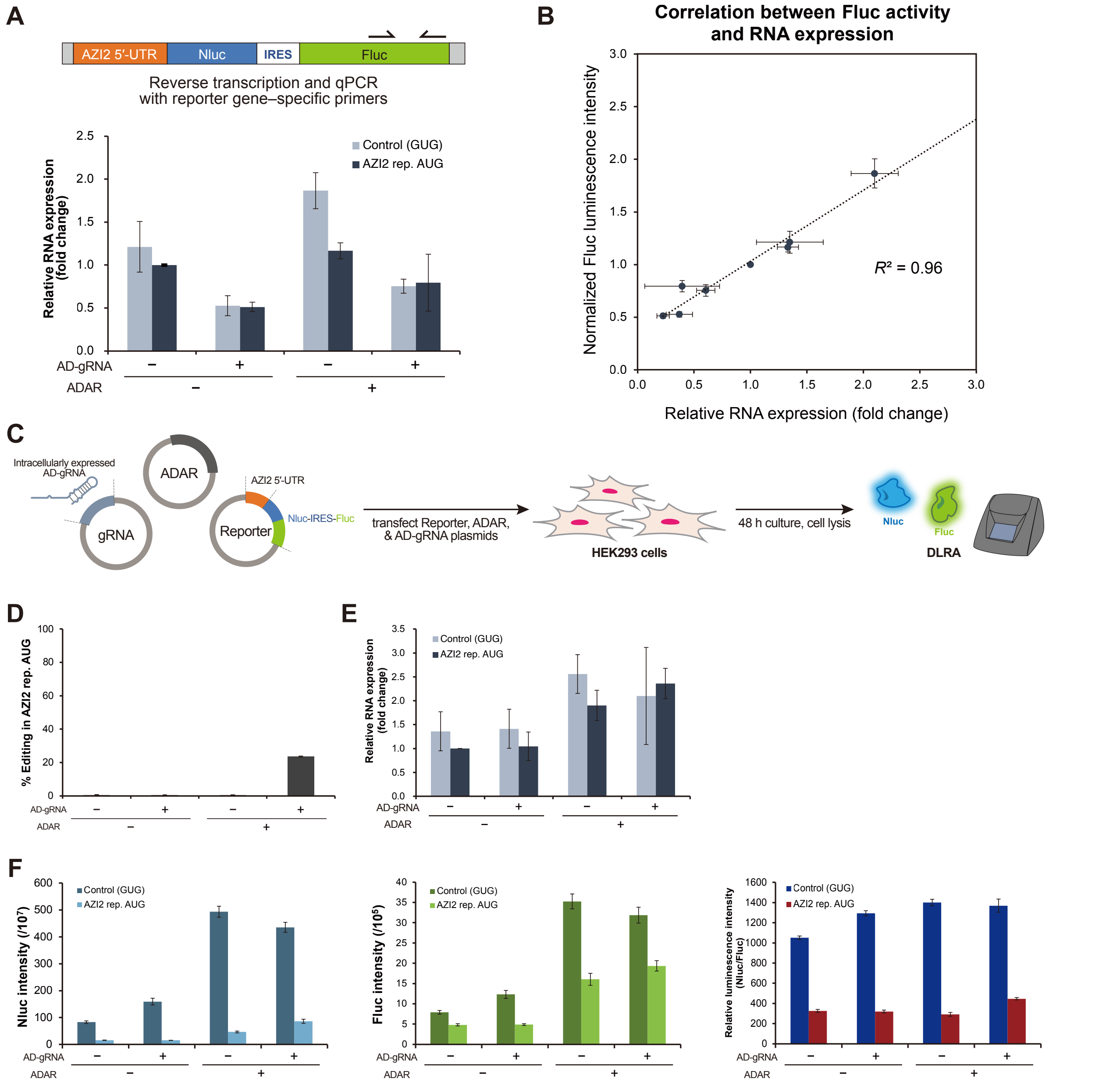

(A) qPCR analysis of the AZI2 rep. AUG reporter under each editing condition corresponding to Fig. 4 and Fig. S5. When chemically modified AD-gRNAs were used, an AD-gRNA–dependent decrease in reporter RNA abundance was observed. Values were normalized to GAPDH mRNA levels, and relative RNA expression was calculated using the 2^－ΔΔCt method.

(B) Scatter plot showing the correlation between Fluc luminescence and reporter RNA abundance. The y-axis represents Fluc luminescence values normalized from the AZI2 reporter data shown in Fig. S5, and the x-axis represents the relative RNA levels quantified in panel (A). The coefficient of determination (R^2), calculated from the fitted regression curve, is indicated within the plot.

(C) Schematic of the intracellular AD-gRNA expression system. The stem-loop–structured AD-gRNA used for *in vitro* assays was cloned into the pSUPER neo vector and co-transfected with ADAR and reporter plasmids to avoid chemical modifications that may destabilize RNA.

(D) Editing efficiencies obtained using intracellularly expressed AD-gRNA and ADAR p110_E713Q. Approximately 20% editing was achieved under ADAR(+)/AD-gRNA(+) conditions, as determined by Sanger sequencing peak area ratios.

(E) qPCR analysis of the AZI2 rep. AUG reporter under the experimental conditions corresponding to panels (C) and (F). When intracellularly expressed AD-gRNAs were used, no reduction in reporter RNA abundance was observed, in contrast to the decrease detected with chemically modified AD-gRNAs. Values were normalized to GAPDH mRNA levels, and relative RNA expression was calculated using the $2^{-\Delta\Delta Ct}$ method.

(F) Raw luminescence values of the AZI2 reporter constructs (Nluc and Fluc) measured under the editing conditions shown in panel (E). These raw values were used to calculate relative Nluc activity, which is presented in the right panels.

Data are shown as mean ± SD (n = 3 independent biological experiments; independent transfections processed separately). Source data are provided as a Source Data file.

Supplementary Figure 7. Demonstration of translational upregulation by targeting the 5′-UTR of CNOT3, a non-endogenous RNA editing substrate, supporting the generalizability of uORF-based translational regulation

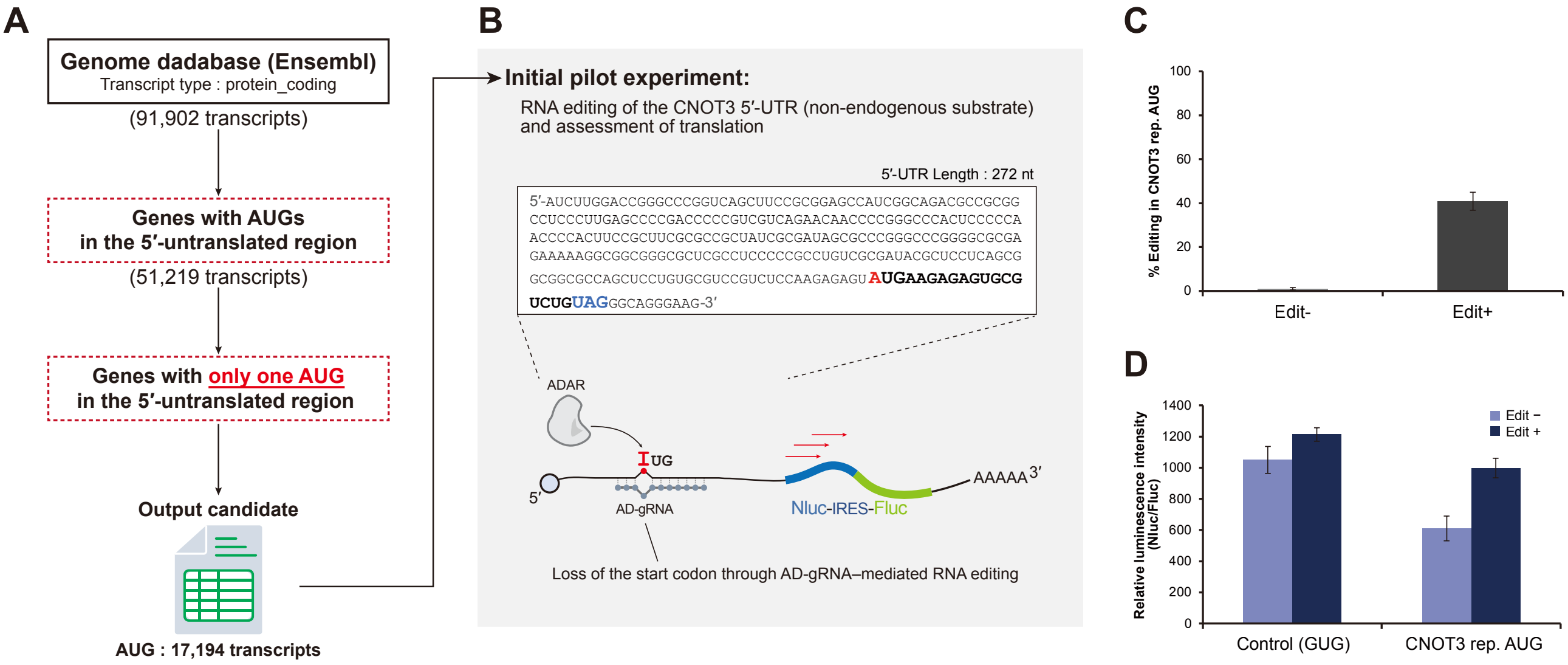

(A) Identification of candidate transcripts potentially amenable to translation upregulation via uORF elimination. Among 91,902 Ensembl transcripts analyzed, 17,194 transcripts contained exactly one AUG codon in the 5′-UTR, representing candidates in which RNA editing (AUG→IUG) could remove a repressive uORF.

(B) Schematic overview of the reporter assay used to evaluate editing-dependent translational regulation of the CNOT3 5′-UTR. The reporter encodes Nluc followed by an IRES-Fluc cassette, allowing normalization of translation to RNA abundance.

(B) Schematic overview of the reporter assay used to evaluate editing-dependent translational regulation of the CNOT3 5′-UTR. As in previous assays, the CNOT3 5′-UTR was fused upstream of an Nluc–IRES–Fluc reporter cassette. Introduction of an AD-gRNA targeting the single endogenous AUG codon within the CNOT3 5′-UTR induced its conversion to IUG, thereby eliminating the uORF created by this start codon.

(C) Editing efficiency at the target site within the CNOT3 5′-UTR under conditions without editing components (Edit–) and with co-expression of ADAR2_E488Q and the corresponding AD-gRNA (Edit+). Sanger sequencing analysis confirmed 40.9% editing at the designed target site.

(D) Relative Nluc luminescence of the CNOT3 reporter under the conditions described in (C). A ~1.6-fold increase in Nluc activity was observed upon editing-dependent removal of the uAUG, demonstrating translational upregulation comparable to that seen with endogenous editing-substrate reporters.

Data are shown as mean ± SD (n = 4 independent biological experiments; independent transfections processed separately). Source data are provided as a Source Data file.

Supplementary Figure 8. Sanger sequencing chromatograms of 5′-UTR editing under the conditions described in Fig. 4

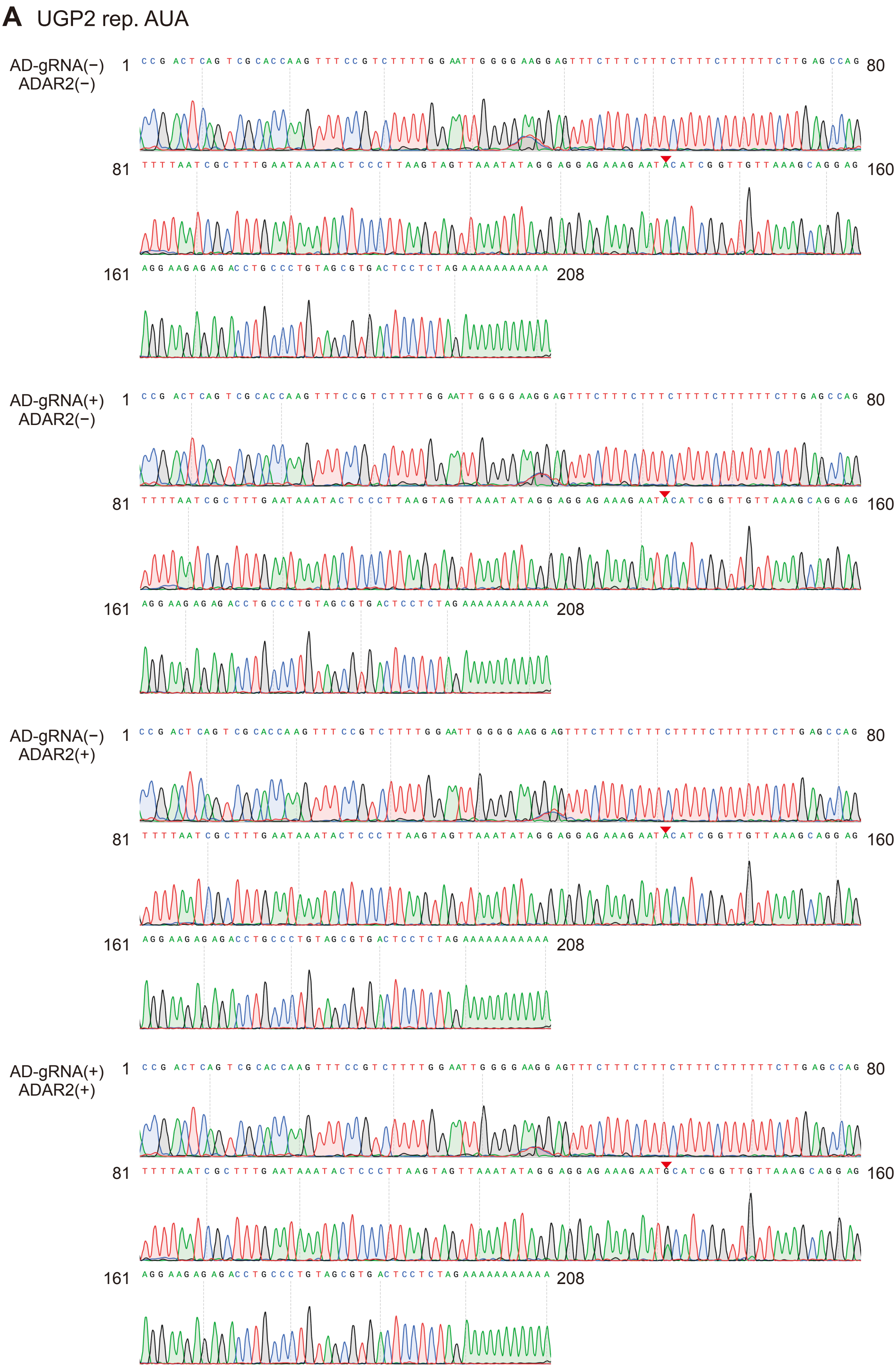

B ASF1A rep. AUG

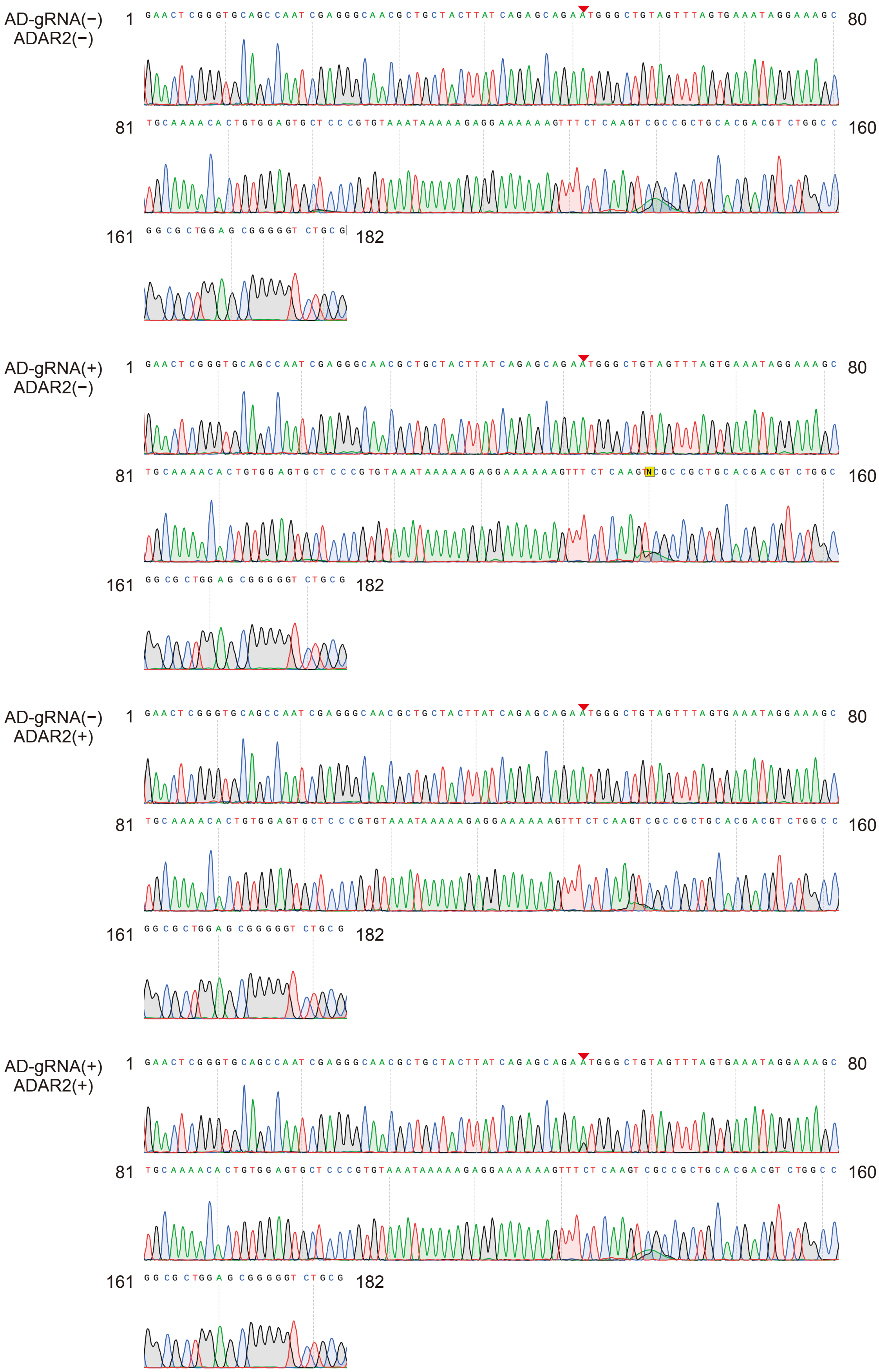

C AZI2 rep. AUG

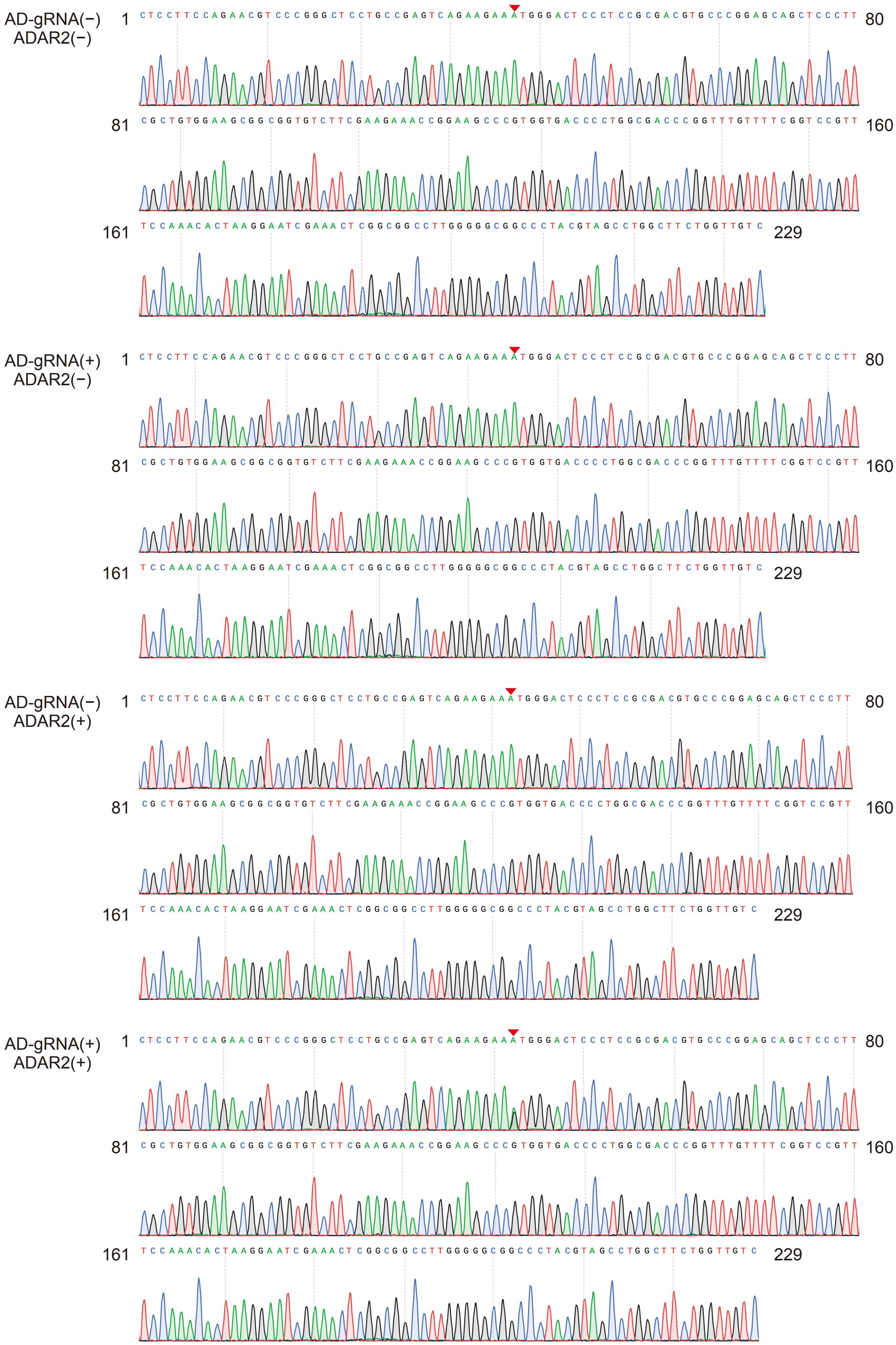

Representative chromatograms are shown for (A) UGP2 rep. AUA, (B) ASF1A rep. AUG, and (C) AZI2 rep. AUG. Red triangles above the sequences indicate the AD-gRNA–targeted editing sites.
